# Notum regulates the cusp and root patterns in mouse molar

**DOI:** 10.1038/s41598-024-64340-w

**Published:** 2024-06-13

**Authors:** Dinuka Adasooriya, Ju-Kyung Jeong, Minjae Kyeong, Shiqi Kan, Jiwoo Kim, Eui-Sic Cho, Sung-Won Cho

**Affiliations:** 1 Division of Anatomy and Developmental Biology, Department of Oral Biology, BK21 FOUR Project, Yonsei University College of Dentistry, Seoul, Korea; 2 Cluster for Craniofacial Development and Regeneration Research, Institute of Oral Biosciences, Jeonbuk National University School of Dentistry, Jeonju, Korea

**Keywords:** Notum protein, Molar, Tooth crown, Tooth root, Wnt signaling, Morphogenesis, Developmental biology, Morphogenesis, Body patterning

## Abstract

*Notum* is a direct target of Wnt/β-catenin signaling and plays a crucial role as a Wnt inhibitor within a negative feedback loop. In the tooth, *Notum* is known to be expressed in odontoblasts, and severe dentin defects and irregular tooth roots have been reported in *Notum*-deficient mice. However, the precise expression pattern of *Notum* in early tooth development, and the role of *Notum* in crown and root patterns remain elusive. In the present study, we identified a novel *Notum* expression in primary enamel knot (EK), secondary EKs, and dental papilla during tooth development. *Notum*-deficient mice exhibited enlarged secondary EKs, resulting in broader cusp tips, altered cusp patterns, and reduced concavity in crown outline. These alterations in crown outline led to a reduction in cervical tongue length, thereby inducing root fusion in *Notum*-deficient mice. Overall, these results suggest that the secondary EK size, regulated by the Wnt/Notum negative feedback loop, has a significant impact on the patterns of crown and root during tooth morphogenesis.

## Introduction

*Notum*, identified as a palmitoleoyl-protein carboxylesterase, functions both as a target and modulator within the Wnt/β-catenin signaling pathway. Wnts undergo O-linked palmitoleoylation at a conserved serine, necessary for binding to Fzd receptors. *Notum* removes the O-linked palmitoleate modification, thereby deactivating Wnts^1^. Notum plays a pivotal role in tracheal patterning, neural and head development, and bone and intestine homeostasis by downregulating Wnt/β-catenin signaling in a negative feedback loop^2–4^.

A recent study reported dentin defects and irregular, short roots in *Notum-*deficient mice^5^. Subsequent investigations revealed the expression of *Notum* specifically in the progenitors of odontoblasts adjacent to Hertwig’s epithelial root sheath, and single-cell RNA sequencing analyses also reported *Notum* expression in early odontoblasts near the cervical loop during root formation^6,7^. While it has been reported that crown shape and enamel remain normal across all molars^5^, a comprehensive analysis of crown morphology was lacking. The precise *Notum* expression pattern in early tooth development and its role in crown patterning remains elusive.

In the present study, we investigated the gene expression pattern of *Notum* using RNA in situ hybridization and single-cell RNA sequencing analysis. Furthermore, we explored the roles of *Notum* in tooth morphogenesis by examining the phenotypic changes in cusp patterns, crown outlines, cervical tongue patterns, and root patterns through geometric morphometric analysis of molars in *Notum*-deficient mice. We found a notable increase in the size of secondary enamel knot (EK) in *Notum*-deficient mice and demonstrated the interconnected nature of sequential processes from secondary EK to root pattern in molars of *Notum*-deficient mice.

## Materials and methods

All methods, including the animal experiments, were approved by the Yonsei University Health System Institutional Animal Care and Use Committee (YUHS-IACUC) under the approval number 2020−0279. All the procedures were performed in accordance with the guidelines and regulations of this committee and the Animal Research: Reporting In Vivo Experiments (ARRIVE 2.0) guidelines.

### Animals

F0 *Notum* knockout mouse (*Notum*^*em*1*(IMPC)Tcp*^) generated on the C57BL/6N background strain were purchased from the international mouse phenotyping consortium (IMPC) (www.mousephenotype.org) and mated with C57BL/6N wild type mice to get the F1. F2 and above generations of mice (*Notum*^+*/*+^ and *Notum*^*−/−*^) were used for the study after euthanization utilizing CO_2_ exposure at selected postnatal ages and embryonic days. A mix of males and females was assigned without considering the sex, and at least one individual from each sex was included in each group after screening for the genotype with PCR. Approximately 90% of *Notum*^*−/−*^ mice display perinatal lethality.

### Single-cell RNA sequencing analysis of publicly available datasets

Publicly available scRNA-seq datasets of E14.5 and E16.5 mice molar were retrieved from the Gene Expression Omnibus (GEO) under the accession numbers GSE189381^8^ and GSE162413^9^. These datasets were available as pre-processed count matrices. The single-cell RNA-seq datasets were processed, explored, and visualized using Cellenics® community instance (https://scp.biomage.net/) hosted by Biomage (https://biomage.net/).

Pre-filtered count matrices were imported into the Cellenics®, and the barcodes were sequentially filtered using the automatic filtering settings in the four filtering steps: cell size distribution filter, mitochondrial content filter, number of genes vs UMI filter, and doublet filter. After the filtering and QC, a total of 30,744 cells for E14.5 and 29,307 for E16.5 were included in the final visualization and clustering. The two E14.5 datasets and the E16.5 datasets were separately integrated with the "Harmony" method and normalized with the "LogNormalize" function. The top 2000 highly variable genes (HVGs) were selected using the variance stabilizing transformation (VST) technique. Dimensionality reduction is performed to summarize and visualize the data with Principal-component analysis (PCA) with 30 Principal Components (PCs) for both E14.5 and E16.5, explaining over 90% of the total variation within the datasets. Cell clusters were generated with the Louvain method and visualized using Uniform Manifold Approximation and Projection (UMAP) embedding at a resolution of 0.3. All cell clusters were manually annotated using the available literature^8,9^.

### Whole-mount RNA in situ hybridization

Tooth germs were dissected from the mandibles and maxillae of *Notum*^+*/*+^ and *Notum*^*−/−*^ mice at E14.5, E16.5, E17.5, and E18.5 on DEPC-PBS and fixed in 4% PFA in DEPC-PBS overnight at 4 °C. To facilitate the permeabilization, they were treated with 10 µg/ml proteinase K for 45 min at room temperature. The hybridization was performed with digoxigenin-labeled *Fgf4,* and *Notum* RNA probes 1 µg/ml in hybridization solution for 20 h at 67 °C to 70 °C. Specimens were equilibrated with color reaction buffer containing Tris, MgCl_2_, NaCl, Tween 20, and 4-nitro-blue-tetrazolium (NBT)/5-bromo-4chloro-3-indolyl-phosphate (BCIP) (Roche, USA). When the appropriate color was developed, samples were washed with PBS and post-fixed with 4% PFA. Images were taken using a Leica S9D microscope equipped with a Leica M170 HD digital camera.

### Section in situ hybridization

Mandibles were dissected from E14.5 and E16.5 wild-type mouse embryos on the cold DEPC-PBS and fixed with 4% PFA in DEPC-PBS overnight at 4 °C with rocking. Samples were rinsed in DEPC-PBS and decalcified with the 10% EDTA in DEPC-PBS at 4 °C for 4 days. The decalcified samples were washed with DEPC-PBS followed by a saline wash, and dehydration began with Saline: Ethanol 1:1 solution followed by 70%, 80%, and 90% Ethanol with DEPC and 100% ethanol. Samples were cleared in xylene, followed by wax infiltration, and embedded in paraffin wax. Paraffin-embedded samples were sectioned by 5 µm, and sections were collected on glass slides. An antisense *Fgf4, Shh, Bmp4,* and *Notum* RNA probes were designed and produced by Advanced Cell Diagnostics (Newark, USA). In situ hybridization was performed with the RNAscope® 2.5 High Definition (HD) assay-brown (Advanced Cell Diagnostics) according to the user manual 322,452 (FFPE sample preparation and pretreatment) and 322,310 (RNAscope® 2.5 HD Detection Reagent-brown user manual) provided by the manufacturer. The following RNA probes were used: (1) Mm-Fgf4 (514,311, targeting NM_010202.5, nucleotide 313–1486) (2) Mm-Shh (314,361, targeting NM_009170.3, nucleotide 307–1197) (3) Mm-Bmp4 (401,301, targeting NM_007554.2, nucleotide 586–1673), (4) Mm-Notum (428,981, targeting NM_175263.4, nucleotide 406–1623), (5) Mm-Lef1 (44,186, targeting NM_010703.4, nucleotide 1361–2354). Images were taken using an Olympus BX43 microscope equipped with an Olympus DP23 digital camera.

### Micro-computed tomography and geometric morphometric analysis

Mouse hemimaxilla and hemimandibles (*Notum*^+*/*+^, *Notum*^*−/−*^ at PN14, PN35) were fixed in 4% Paraformaldehyde (PFA) in PBS. Micro-computed tomography (micro-CT) images were obtained using a micro-CT scanner (Skyscan1173, Bruker, Belgium) at 130 kV and 60 µA alongside 0.25 g/cm^3^ and 0.75 g/cm^3^ Phantom rods. Micro-CT data were reconstructed using NRecon (Version 1.6) with consistent parameters. Skull and tooth micro-CT images were converted to 3D volumes using the software 3D Slicer (Version 4.1, http://www.slicer.org) and OnDemand 3D (Version 1.0, Cybermed, Korea). Eight and seven landmarks are placed on the cusps of the 3D-reconstructed maxillary and mandibular first molars (M1), respectively, and 64 equally distanced landmarks are placed along the crown outlines of the maxillary and mandibular M1 occlusal view 2D captures (PN14 n = 10, and PN35 n = 12 per group) (Fig. 3A, G) using the software Blender (Version 3.2.1, Blender Foundation, Netherlands). Principal component (PC) analysis and discriminant function (DF) analysis with leave-one-out cross-validation were performed on Procrustes shape coordinates to define shape features using the software MorphoJ (Version 1.07a, Klingenberg lab, University of Manchester, UK).

### Scanning electron microscopy

Scanning electron microscopy (SEM) was performed on the PN35 molars of *Notum*^+*/*+^ and *Notum*^*−/−*^ mandibles and maxillae (n = 3 per group). Mandibles and maxilla samples were fixed overnight in 4% PFA in PBS. The samples were dehydrated using ethanol series, air-dried, fixed for 2 h in 1% OsO_4_, and dried with a freeze dryer (ES-2030, Hitachi, Japan). Mandibular and maxillary molar with the alveolar bone were mounted on metallic stubs and platinum coated to a thickness of 100 nm using an ion coater (E-1010, Hitachi) and imaged under the scanning electron microscope (S-3000N, Hitachi).

### Whole mount immunohistochemistry

Maxillary and mandibular molar tooth germs were isolated from *Notum*^+*/*+^ and *Notum*^*−/−*^ mice at PN0 and PN7 (n = 5 per group) (see supplementary Fig. 9A−T1 online). They were dissected in PBS and fixed in Methanol/DMSO (4:1) at 4 °C overnight and then in Methanol/DMSO/H_2_O_2_ (4:1:1) at 4 °C overnight. Samples were stored in 100% methanol at − 20 °C. The tooth germs were rehydrated with 50% Methanol in PBS, followed by PBS and then PBSMT. Next, the primary antibody (Human/Mouse E-Cadherin Antibody, R&D Systems) 1:200 diluted in PBSMT was added and incubated overnight at 4 °C. The next day, the samples were washed five times with PBSMT for 1 h each. The secondary antibody (Donkey anti-Goat IgG (H + L) Secondary Antibody, HRP, Invitrogen) 1:500 diluted in PBSMT was added and incubated overnight at 4 °C. The following day, samples were washed five times with PBSMT for 1 h each. The color reaction was performed using the DAB chromogen kit (Liquid DAB + Substrate Kit for Immunohistochemistry, GBI Labs) as described in the manufacturer's manual. Two drops of DAB chromogen were diluted in 1 ml of DAB substrate buffer, and the tooth germs were incubated in an enclosed chamber at room temperature until the appropriate color was developed. Samples were washed with distilled water and post-fixed with 4% PFA and imaged using a Leica S9D microscope equipped with a Leica M170 HD digital camera.

### RNA sequencing

Maxillary and mandibular molar tooth germs were dissected from *Notum*^+*/*+^and *Notum*^*−/−*^ littermate mouse embryos at E14.5 and E16.5 (n = 3 per biological replicate, and 2 replicates per group) in DEPC-PBS, transferred to RNAlater® solution (Life Technologies, Thermo Fisher Scientific, USA), and stored at − 20 °C. Tooth germs were homogenized in TRIZOL® (Invitrogen) with the 0.5 mm stainless steel beads using the Bullet Blender® homogenizer (Next Advance, USA). Total RNA was phase separated with chloroform and precipitated with isopropyl alcohol. The RNA pellet was washed with 75% ethanol and eluted with RNase-free water. RNA concentration and quality were assessed using RNA ScreenTape® (Agilent Technologies, Germany).

The library was prepared and sequenced on an Illumina platform, and paired-end reads were generated. After the quality control, reads were mapped into the reference genome (Mm10). The read numbers mapped to each gene were counted with featureCounts v1.5.0-p3, and the FPKM of each gene was calculated based on the length of the gene and read count mapped to the genes. Differential expression analysis of two groups (two biological replicates per condition) was performed on the read counts using the DESeq2 R package (1.20.0). The resulting P values were adjusted using the Benjamini & Hochberg method. A corrected *P*-value of 0.05 was assigned as the threshold for significantly differential expression.

### Measurement of the cusp tip area, root length, mesiodistal and buccolingual widths, and statistical analysis

The cusp tip area was measured in micro-CT sections of PN14 maxillary and mandibular M1 molars of *Notum*^*−/−*^ and *Notum*^+*/*+^ mice (n = 10 per group). The area of the cusp tip was measured 0.1 mm below the cusp tip point perpendicular to the cervical plane (see supplementary Fig. 7A−B online). The root length was measured in 3D segmented PN35 maxillary and mandibular M1 of *Notum*^*−/−*^ and *Notum*^+*/*+^ mice (n = 10 per group) (see supplementary Fig. 7C−D online). The mesiodistal and buccolingual widths were measured in 3D segmented PN35 maxillary M1 (n = 12) and mandibular M1 (n = 12) in *Notum*^*−/−*^ and *Notum*^+*/*+^ mice. Reference points were made at the most mesial and distal ends in the mesiodistal axis of the crown and the most lingual and buccal points in the buccolingual axis. Then, a three-dimensional straight line connecting two points at each axis was created, and the distance was measured.

The cusp tip area and the root length were presented as bar charts with error bars indicating the standard deviation (SD). The crown width, interradicular distance, and interradicular area data were expressed as box and whisker plots based on 12 M1 samples per group. The Mann–Whitney U test was applied for the pairwise statistical comparisons between groups, and linear regression was applied to analyze the relationship between the dimensions using the SPSS software (version 26.0; IBM Corp., USA). *P* value < 0.05 was considered a statistically significant difference.

## Results

### *Notum* was expressed in primary EK, secondary EKs, and dental papilla

**Figure 1:**
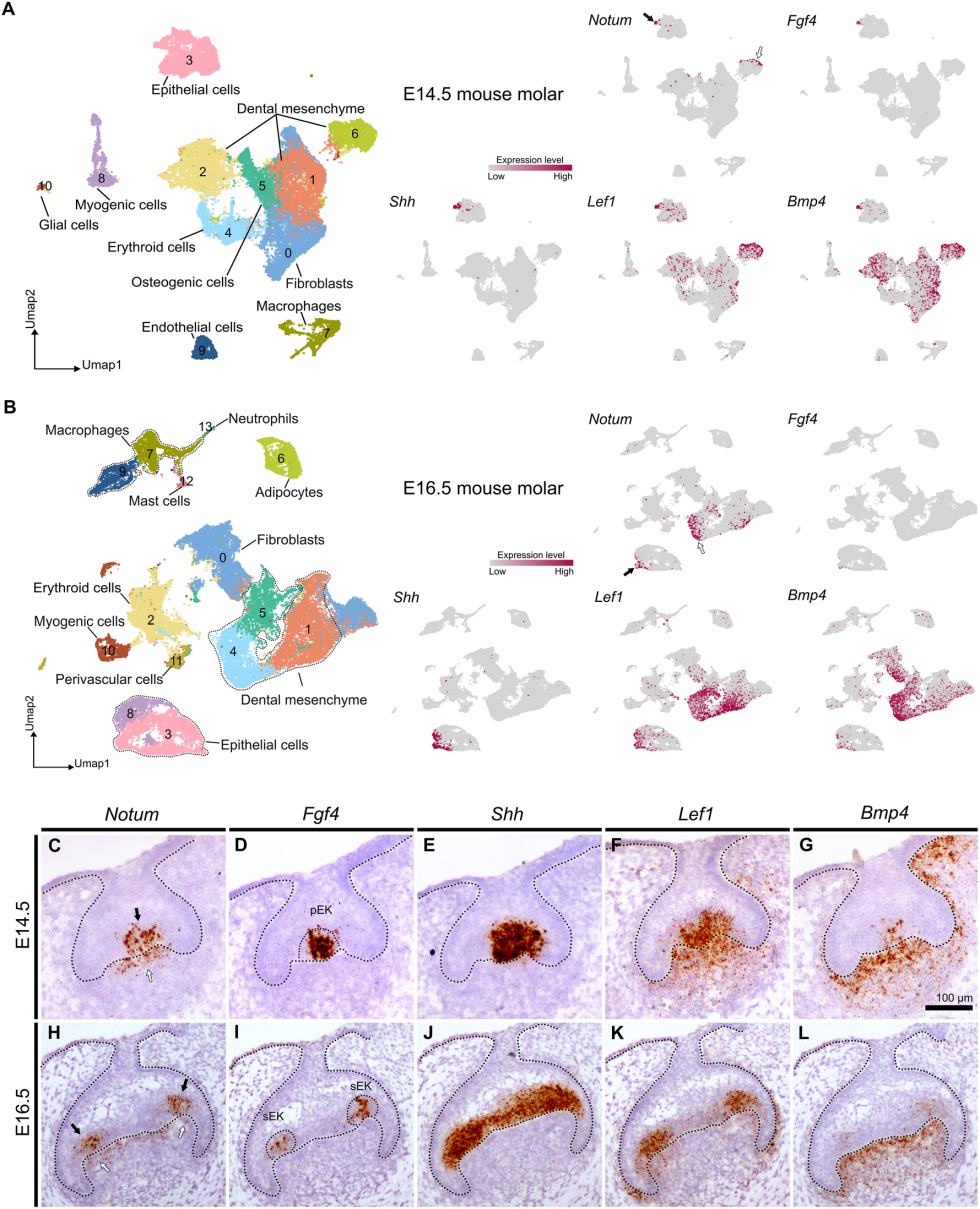
Gene expression pattern of *Notum* in developing molars at embryonic day (E)14 and E16 mouse embryos. (**A**,** B**) Single-cell RNA sequencing analysis of E14.5 and E16.5 molars depicts *Notum* expression with other known enamel knot (EK) marker genes (*Fgf4, Shh, Lef1* and *Bmp4*). Epithelial cells expressing *Notum* colocalize with cells expressing *Shh, Fgf4, Bmp4,* and *Lef1* at both stages (black arrows in A and B), while mesenchymal cells expressing *Notum* (white arrows) also express *Lef1* and *Bmp4*. **(C−L)** In the frontal sections of mandibular molars at E14.5 and E16.5, *Notum* is expressed in the primary EK (pEK) at E14.5, secondary EK (sEK) at E16.5, and in the thin outer layer of the dental papilla. *Fgf4* expression is observed at the primary EK at E14.5 and secondary EKs at E16.5. *Shh* is expressed in the primary enamel knot at E14.5, and in the inner dental epithelium at E16.5. *Lef1* and *Bmp4* are expressed in the primary EK at E14.5, secondary EKs at E16.5, and dental papilla. Scale bar, 100 µm.

To investigate the expression pattern of *Notum* during tooth development, we conducted cell clustering analysis in molars at E14.5 and E16.5, utilizing recently published single-cell RNA sequencing data sets^8,9^. The UMAP illustration showed unbiased identification of 11 clusters at E14.5 and 14 clusters at E16.5 molars (Fig. 1A, B). The differentially expressed genes in each cluster were listed in the cluster map and heatmap (see Supplementary Fig. 1−4 online). *Notum* expression was identified in a small subset of both epithelial and dental mesenchymal clusters at both E14.5 and E16.5. In the map (Fig. 1A, B) and frontal sections of the mandibular first molar (M1) (Fig. 1C−L), epithelial cells expressing *Notum* colocalized with *Shh, Fgf4, Bmp4,* and *Lef1*. Specifically, *Notum* expression in epithelial cells was localized in primary EK at E14.5 and secondary EKs at E16.5, aligning with the expression pattern of *Fgf4*, one of the EK markers. This specific expression of *Notum* within EK suggests a contributory role of *Notum* in the intricate process of cusp patterning. On the other hand, mesenchymal cells expressing *Notum* were co-localized with *Lef1* and *Bmp4* at E14.5 and E16.5 in the cluster map (Fig. 1A, B) and in sections of the mandibular M1 (Fig. 1C−L). *Notum* was expressed in a thin outer layer of dental papilla in the section (Fig. 1C, H). At E14.5 and 16.5, *Notum* was expressed in both the epithelium and mesenchyme at the cusp region. As tooth development advances, *Notum* expression persisted exclusively in the mesenchyme but diminished in the epithelium (see Supplementary Fig. 5 online).

## *Notum*^*−/−*^ mice displayed abnormalities in both crown and root morphology

**Figure 2:**
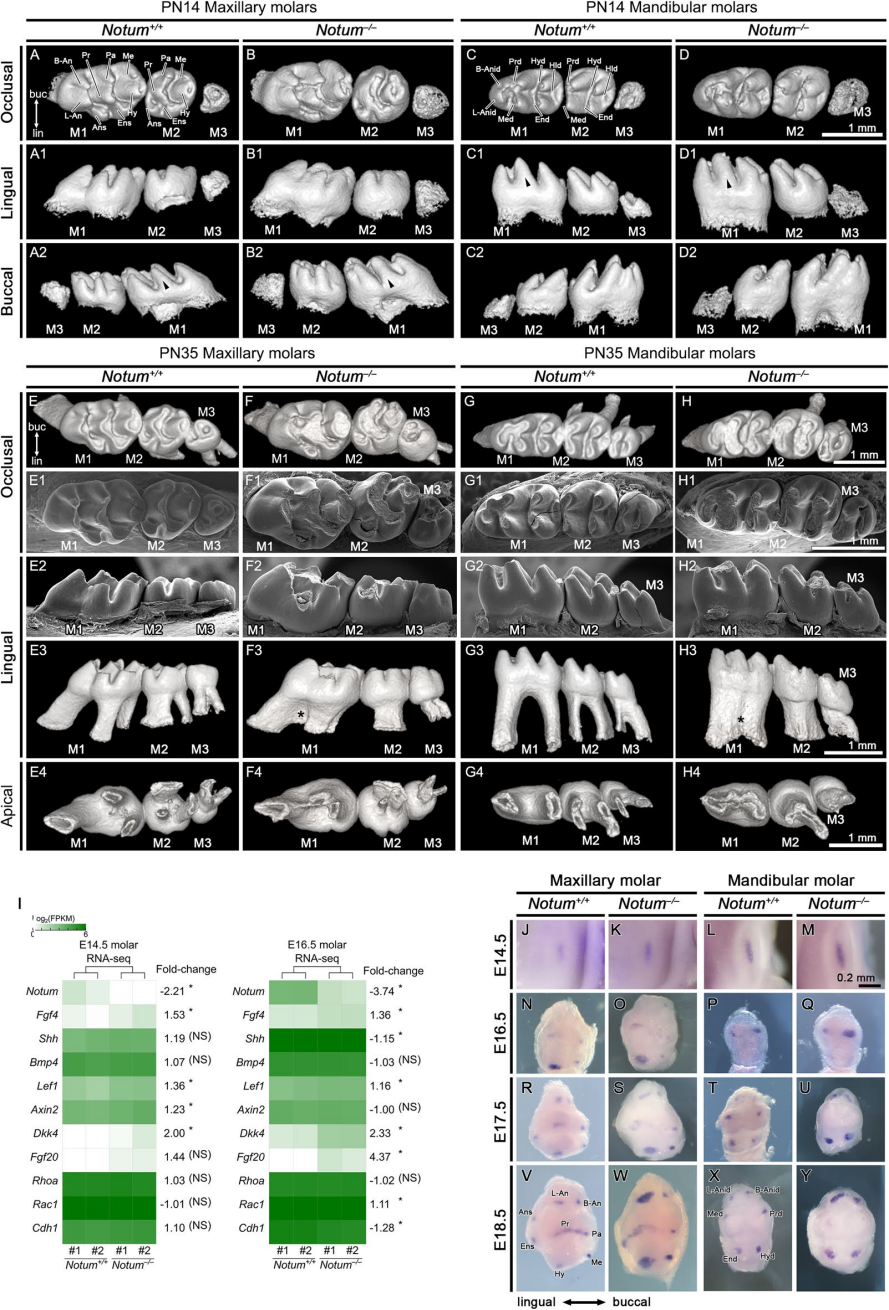
Morphological changes in crown and root of maxillary and mandibular molars in *Notum*^*−/−*^ mice at postnatal day (PN) 14 and PN35. (**A−D2**) At PN14, first molar (M1), second molar (M2), and third molar (M3) appear slightly larger in *Notum*^*−/−*^ mice compared to *Notum*^+*/*+^ mice. Broader cusp tips are observed in *Notum*^*−/−*^ molars in both occlusal view and lateral views, particularly in the paracone of maxillary M1 (arrowheads in A2 and B2) and metaconid in mandibular M1 (arrowheads in C1 and D1). (**E−H4**) At PN35, differences in crown outline and size persist, with *Notum*^*−/−*^ molars showing severe attrition. 3D-reconstructed molars reveal root fusion in maxillary and mandibular M1 of *Notum*^*−/−*^ mice in lingual and apical views. (**I**) Heat maps displaying the alterations in the expression level of selected genes in RNA-sequencing analysis of the E14.5 and E16.5 molars of *Notum*^+*/*+^ and *Notum*^*−/−*^ mice. *Notum* is significantly downregulated in *Notum*^*−/−*^ mice, while *Dkk4* and *Fgf20* are significantly upregulated at E16.5. *Fgf4* and *Lef1* show slight upregulation at both E14.5 and E16.5. Cell adhesion-related genes *Rac1* and *Cdh1* exhibit slight upregulation and downregulation, respectively, at E16.5. (**J−Y**) Expression of *Fgf4* in developing maxillary M1 and mandibular M1. *Fgf4* is expressed in the primary EK of maxillary and mandibular M1 at E14.5 and in secondary EKs from E16.5. At E16.5, E17.5, and E18.5, M1s show the enlarged secondary EKs expressing *Fgf4* in *Notum*^*−/−*^ mice compared to *Notum*^+*/*+^ mice in maxilla and mandible. B-Anid: buccal anteroconid, L-Anid: lingual anteroconid, Prd: protoconid, Med: metaconid, Hyd: hypoconid, End:entoconid, Hld: hypoconulid. B-An: buccal anterocone, L-An: lingual anterocone, Ans: anterostyle, Pa: paracone, Pr: protocone, Ens: enterostyle, Me: metacone, Hy: hypocone, lin: lingual, buc: buccal. Scale bars in A**−**H4, 1 mm and J**−**Y, 0.2 mm. * P < 0.05, NS: non-significant.

To investigate the role of *Notum* in tooth morphology, we examined morphological changes in crown and root in PN14 molars before occlusal attrition. Notably, *Notum*^*−/−*^ mice exhibited broader tips in most cusps of both M1 and second molar (M2) (Fig. 2A–D2, Supplementary Fig. 6, supplementary Fig. 7A−B online). However, the cusp base dimension did not show a notable change in *Notum*^*−/−*^ mice. In *Notum*^*−/−*^ mice, half of maxillary M1s displayed fusion between anterostyle and enterostyle (see Supplementary Fig. 6H−L online), and many of mandibular M1s showed fusion among lingual anteroconid, buccal anteroconid, and protoconid (see Supplementary Fig. 6R−X online).

The root pattern was changed in PN35 molars. Consistent with a previous study^5^, we observed shorter molar roots and severe occlusal wear in *Notum*^*−/−*^ mice compared to *Notum*^+*/*+^ mice (Fig. 2E−H4, supplementary Fig. 7C−D online). While the earlier study characterized the root pattern of *Notum*^*−/−*^ mice as irregular, our finding revealed root fusion in both the maxillary and mandibular molars of *Notum*^*−/−*^ mice (Fig. 2E3−E4, F3−F4, G3−G4, H3−H4, Supplementary Fig. 8 online). No morphological differences were observed between *Notum*^+*/−*^ and *Notum*^+*/*+^ molars.

## In* Notum*^*−/−*^ mice, the size of secondary EKs was notably enlarged

To investigate the molecular function of *Notum* during tooth development, we conducted RNA sequencing analysis on E14.5 and E16.5 molars. We identified 131 and 424 genes with expression level changes exceeding two-fold in E14.5 and E16.5 molars, respectively (see Supplementary Table 1−4 online). Notably, the expression level of *Fgf20* and *Dkk4*, known as EK-specific genes, were significantly elevated in *Notum*^*−/−*^ molars at E16.5. *Fgf20* and *Dkk4* ranked as the top two and sixteenth, respectively, in the list of upregulated genes (Supplementary Table 1 online, Fig. 2I). Furthermore, the expression level of *Fgf4* was slightly increased in both E14.5 and E16.5 *Notum*^*−/−*^ molars. *Dkk4, Fgf4,* and *Fgf20* are recognized as target genes of the Wnt/β-catenin signaling pathway. The expression levels of *Lef1, Bmp4,* and *Axin2*, other Wnt target genes expressed in both EK and dental papilla, were not significantly changed in *Notum*^*−/−*^ molars (Fig. 2I).

To elucidate the size and spatial pattern of secondary EKs in *Notum*^*−/−*^ mice, we examined the pattern of *Fgf4* expression, a reliable marker for both primary and secondary EKs. In *Notum*^*−/−*^ mice, each maxillary M1 displayed a primary EK at E14.5, similar to *Notum*^+*/*+^ mice (Fig. 2J−M). At E16.5, *Notum*^*−/−*^ M1 exhibited larger secondary EKs, particularly in protocone and hypocone (Fig. 2N−O). Subsequently, at E17.5 and E18.5, a notable increase in the size of most secondary EKs was observed in *Notum*^*−/−*^ mice, particularly in lingual anterocone and hypocone within M1 (Fig. 2R−S, V−W). In mandibular M1, a substantial enlargement of secondary EKs in protoconid and metaconid at E16.5, and lingual anteroconid, hypoconid, and entoconid was observed in *Notum*^*−/−*^ M1 at E17.5 and E18.5 (Fig. 2P−Q, T−U, X−Y). This increase in secondary EK size appears closely linked to the concurrent dimensional increase in cusp tips of *Notum*^*−/−*^ molars (Fig. 2A−D2). The emergence timing of secondary EKs in maxillary M1 differed between *Notum*^+*/*+^ and *Notum*^*−/−*^ mice. Enterostyle became visible at E17.5 in *Notum*^+*/*+^ M1 (Fig. 2R), while its appearance was delayed until E18.5 in *Notum*^*−/−*^ M1 (Fig. 2S, W). Similarly, anterostyle emerged at E18.5 in *Notum*^+*/*+^ M1 but remained absent until E18.5 in *Notum*^*−/−*^ M1 (Fig. 2V, W).

## *Notum*^*−/−*^ mice displayed changes in cusp patterns and crown outlines

**Figure 3:**
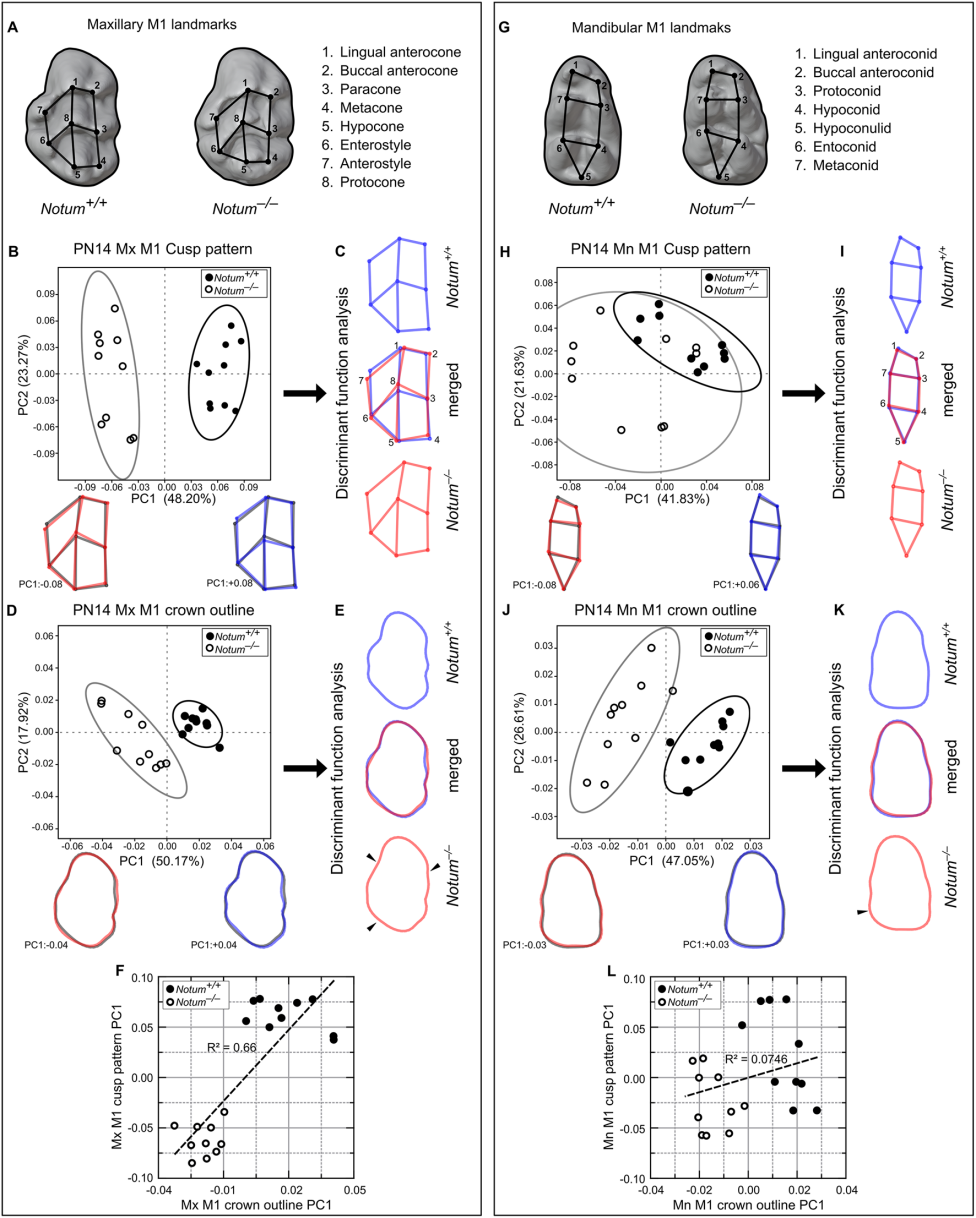
Geometric morphometric changes in cusp pattern and crown outline in *Notum*^*−/−*^ mice at PN14. (**A**, **G**) Cusp landmarks and crown outlines of the maxillary and mandibular first molars (M1). In principal component (PC) analysis, *Notum*^*−/−*^ M1 (white circles) and *Notum*^+*/*+^ M1 (black circles) are plotted along the first two PCs (PC1 and PC2) scores (n = 10 per group). Blue and red wireframes or outlines correspond to positive and negative ends of PC1 axis, respectively. Gray wireframes or outlines indicate the procrustes' mean of all samples along the PC1 axis. In discriminant function (DF) analysis, blue and red wireframes or outlines correspond to *Notum*^+*/*+^ M1 and *Notum*^*−/−*^ M1, respectively (**B−C**) In cross-validated DF analysis, *Notum*^*−/−*^ M1s are correctly classified into *Notum*^*−/−*^ M1 group with a predictive accuracy of 80%, and *Notum*^+*/*+^ M1s are accurately classified into *Notum*^+*/*+^ M1 group with a predictive accuracy of 70%. (**D−E**) In PC analysis of crown outline in maxillary M1, *Notum*^*−/−*^ M1s are clustered separately from the *Notum*^+*/*+^ M1 on PC1 axis. In cross-validated DF analysis, predictive accuracy is 100% for both *Notum*^*−/−*^ M1 and *Notum*^+*/*+^ M1. *Notum*^*−/−*^ M1s show a significant decrease in concavity at both lingual and buccal outlines (arrowheads in E). (**F**) Strong direct relationship between cusp pattern PC1 scores and crown outline PC1 scores in maxillary M1 (R^2^ = 0.66, p = 0.000014). (**H−I**) In PC analysis of cusp pattern in mandibular M1, *Notum*^*−/−*^ M1s are not clustered separately from *Notum*^+*/*+^ M1 along PC1 and PC2 axes. In cross-validated DF analysis, predictive accuracy is 100% for both *Notum*^*−/−*^ M1 and *Notum*^+*/*+^ M1. (**J−K**) In PC analysis of crown outline of mandibular M1, *Notum*^*−/−*^ M1s are clustered separately from *Notum*^+*/*+^ M1s on PC1 axis. In cross-validated DF analysis, *Notum*^*−/−*^ M1s, which also shows a change in distolingual outline (arrowhead in K), are correctly classified into *Notum*^*−/−*^ M1 group and *Notum*^+*/*+^ M1s into *Notum*^+*/*+^ M1 group with a predictive accuracy of 100% and 90%, respectively. (**L**) No relationship between cusp pattern PC1 scores and crown outline PC1 scores in mandibular M1 (R^2^ = 0.0746, P = 0.2481).

To assess the cusp pattern and crown outline differences between *Notum*^*−/−*^ and *Notum*^+*/*+^ M1*,* we performed a three-dimensional (3D) geometric morphometric analysis at PN14 (Fig. 3A−L). Principal component (PC) analysis of maxillary M1 revealed distinct clustering of cusp patterns in *Notum*^*−/−*^ mice, separated from those of *Notum*^+*/*+^ mice on PC plot with PC1. Negative PC1 scores correspond to a cuspal polygon for *Notum*^*−/−*^ M1 (Fig. 3B). In discriminant function (DF) analysis, *Notum*^*−/−*^ M1 displayed distal displacement of anterostyle and enterostyle, buccal displacement of lingual anterocone and buccal anterocone, lingual displacement of hypocone, along with mesial displacement of protocone and metacone from the mean shape (Fig. 3C). PC analysis of the crown outline in maxillary M1 exhibited a clear separation of *Notum*^*−/−*^ M1 from *Notum*^+*/*+^ M1 on PC plot with PC1. Negative PC1 scores correspond to the crown outline of *Notum*^*−/−*^ M1. In DF analysis, *Notum*^*−/−*^ M1 showed reduced concavity in mesiolingual, mesiobuccal, and distolingual outlines (Fig. 3D−E). Cross-validated DF analysis of crown outline consistently distinguished *Notum*^*−/−*^ M1 from *Notum*^+*/*+^ M1. Furthermore, cusp pattern PC1 scores exhibited a strong direct relationship with crown outline PC1 scores (R^2^ = 0.66, *P* = 0.000014) (Fig. 3F).

In mandibular M1, PC analysis of cusp patterns did not show a clear separation between *Notum*^*−/−*^ and *Notum*^+*/*+^ M1 (Fig. 3H). The crown outline in mandibular M1 displayed a distinct separation of *Notum*^*−/−*^ M1 from *Notum*^+*/*+^ M1 on the PC plot with PC1 and PC2 (Fig. 3J). Negative PC1 scores corresponding to the crown outline revealed a buccolingually larger width in *Notum*^*−/−*^ mice. In DF analysis, *Notum*^*−/−*^ M1 showed a considerable change in distolingual outline (Fig. 3J−K). Cross-validated DF analysis of crown outline consistently distinguished *Notum*^*−/−*^ M1 from *Notum*^+*/*+^ M1. In mandibular M1, cusp pattern PC1 exhibited no relationship with crown outline PC1 (R^2^ = 0.0746, *P* = 0.2481) (Fig. 3L).

## The length of cervical tongues was reduced in *Notum*^*−/−*^ mice

**Figure 4:**
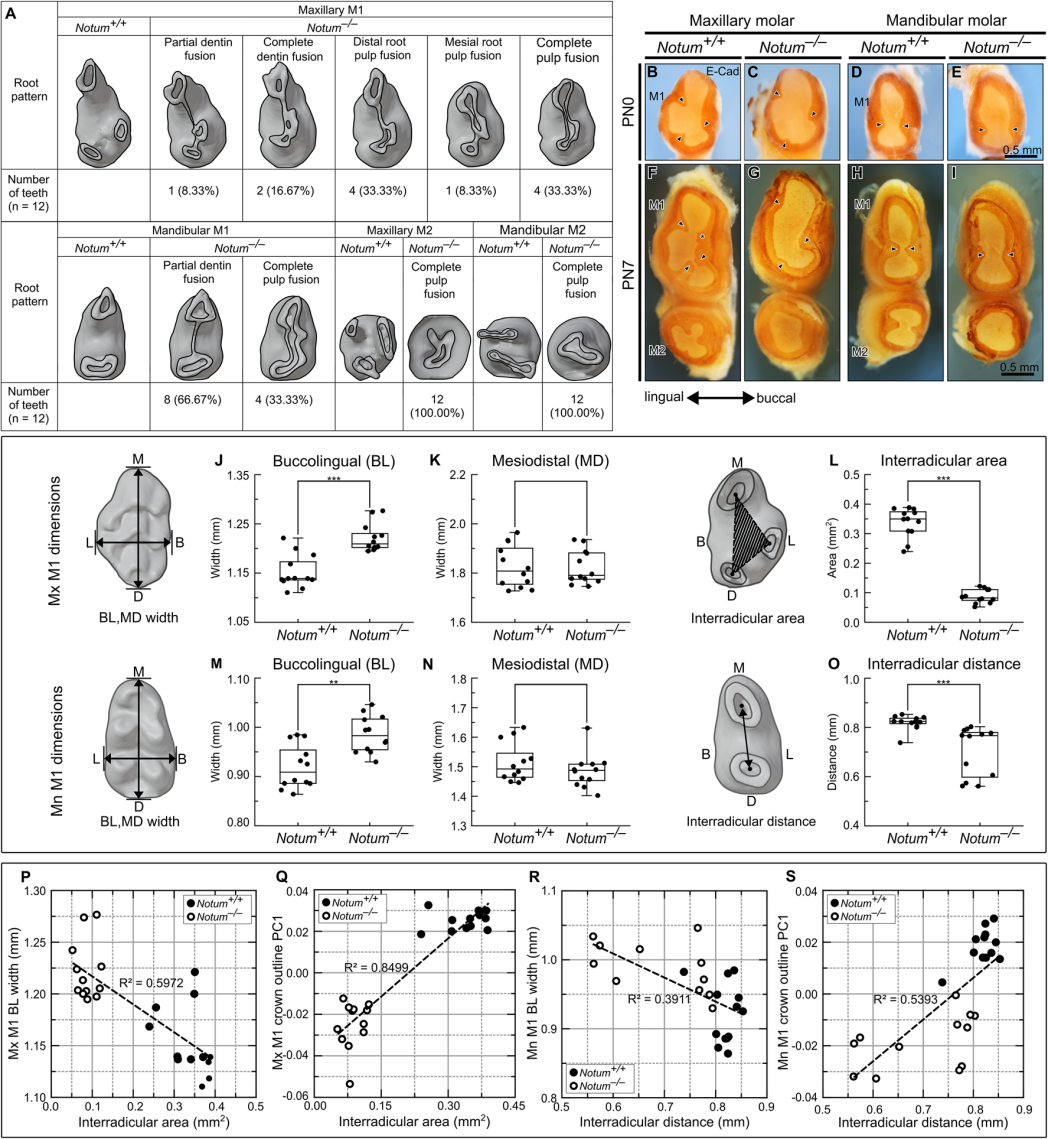
Relationship of crown outline with root pattern in first molar (M1). (**A**) *Notum*^*−/−*^ mice exhibit root fusion across all molars, ranging from partial dentin fusion to complete pulp fusion. (**B−I**) From the apical view of maxillary and mandibular M1 at PN0 and PN7, E-cadherin localization in the epithelium depicts cervical tongue configuration. *Notum*^*−/−*^ M1s display shorter cervical tongues and wider gaps between cervical tongues compared to *Notum*^+*/*+^ M1. (**J−O**) Dimensional changes in crown and root of *Notum*^*−/−*^ M1. *Notum*^*−/−*^ mice show a significant increase in buccolingual width but no change in mesiodistal width in both maxilla and mandible. Maxillary M1 interradicular area is significantly smaller in *Notum*^*−/−*^ mice than *Notum*^+*/*+^ mice, and mandibular M1 interradicular distance is shorter in *Notum*^*−/−*^ mice than *Notum*^+*/*+^ mice. (**P−S**) Relationship between crown outline and root pattern. Linear regression analyses reveal strong relationships between interradicular area dimension and buccolingual width in maxillary M1 (R^2^ = 0.5972, *P*  < 0.001 in T) but weak in mandibular M1 (R^2^ = 0.3911, *P* < 0.001 in V). Strong direct relationship exists between interradicular area dimension and crown outline PC1 scores in both maxillary M1 (R^2^ = 0.8499, *P*  < 0.001 in U) and mandibular M1 (R^2^ = 0.5393, P < 0.001in W). *P  < 0.05, **P < 0.01 and ***P < 0.001. Scale bars, 0.5 mm.

*Notum*^*−/−*^ mice displayed root fusion across all molars. Specifically, in maxillary M1, instances of fusions included partial dentin fusion (incidence = 8.33%), complete dentin fusion (16.67%), distal root pulp fusion (33.33%), mesial root pulp fusion (8.33%), and complete pulp fusion (33.33%). In mandibular M1, analogous observations indicated incidences of partial dentin fusion (66.67%) and complete pulp fusion (33.33%). Notably, every second molar (M2) exhibited complete pulp fusion (Fig. 4A).

To assess the early root patterning, we examined the developmental trajectory of cervical tongues in maxillary and mandibular molars. The configuration of cervical tongues was defined by utilizing the localization of E-cadherin. In *Notum*^*−/−*^ maxillary M1, a notable reduction in length was observed in two lingual cervical tongues, and morphological alteration of a buccal cervical tongue was evident at PN0 and PN7 (Fig. 4B−C, F−G, see supplementary Fig. 9A−J1 online). Similarly, the buccal and lingual cervical tongues showed reduced length at PN0 in *Notum*^*−/−*^ mandibular M1 (Fig. 4D−E, H−I, see supplementary Fig. 9K−T1 online). At PN7 in both maxillary and mandibular M1, the distances between cervical tongues were notably reduced in *Notum*^+*/*+^ mice, contrasting with a considerable distance in *Notum*^*−/−*^ mice (Fig. 4F−I, see supplementary Fig. 9A1–J1, K1−T1 online). This maintenance of distance may potentially hinder the fusion of cervical tongues in *Notum*^*−/−*^ molars.

## The crown outline is closely connected with the root pattern

To explore the connection between crown outline and root patterns, we conducted PC analysis of crown outlines, measured mesiodistal and buccolingual widths of the crown, quantified the extent of root fusion in M1 at PN35, and established a linear regression model between these parameters. PC analysis of the crown outline revealed that *Notum*^*−/−*^ M1 were clustered separately from *Notum*^+*/*+^ M1 on PC plots with PC1, mirroring the pattern observed at PN14 (see supplementary Fig. 10A−D online). The mandibular and maxillary molars of *Notum*^*−/−*^ mice showed an increase in buccolingual width (*p* = 0.000341) while exhibiting no change in mesiodistal width (*p* = 0.908) (Fig. 4J−K, M–N). Quantification of the extent of root fusion by measuring the interradicular area between three roots in maxillary M1 and the interradicular distance between two roots in mandibular M1 indicated a significant decrease in the interradicular area (*P* = 0.000001) and the interradicular distance (*P* = 0.000245) in *Notum*^*−/−*^ M1 (Fig. 4L, O). In linear regression analyses, we found a strong relationship between the maxillary M1 interradicular area and buccolingual width (R^2^ = 0.5972, *P* = 0.000010) and a very strong relationship between the interradicular area and crown outline PC1 scores (R^2^ = 0.8499, *P* = 0.000000) (Fig. 4P−Q). Mandibular M1 showed a weak relationship between interradicular distance and buccolingual width (R^2^ = 0.3911, *P* = 0.001084) and a strong relationship with crown outline PC1 (R^2^ = 0.5393, *P* = 0.000044) (Fig. 4R−S). These findings provide direct evidence of the close relationship between crown outline and root patterns.

## Discussion

Previous studies on *Notum* in tooth primarily focused on the role of *Notum* in odontoblast and its dentin formation^5–7^. In our current study, we investigated *Notum* expression patterns during tooth development and its role in crown and root morphogenesis.

Within the dental papilla, *Notum* expression manifested as a thin outer layer of dental papilla during molar development. The Co-expression of *Notum* with mesenchymal *Lef1* and *Bmp4* suggests a potential association between *Notum* and odontoblast differentiation, given the pivotal roles of *Lef1* and *Bmp4* in this process^10,11^. This finding aligns with prior investigations that delineated the expression pattern of *Notum* in early odontoblasts during postnatal root formation^6,7^. In dental epithelium, *Notum* expression was observed specifically within the primary and secondary EKs. This observation suggests an involvement of *Notum* in cusp pattern formation and crown morphogenesis, given the recognized importance of EKs as regulators shaping the crown by controlling the cusp patterns^12,13^.

The primary EK is a transient signaling center crucial for the bud-to-cap transition of early tooth development^13^. The number and positions of secondary EKs correspond to those of the future tooth cusps^14^. EK size is thought to be regulated by a complex negative feedback loop within intricate signaling networks for EK patterning. Within this loop, Wnts play a pivotal role as the primary activator^15^, extensively documented in tooth and cusp pattern formation^12,16–25^. Since *Dkk4*, acting as both a target and antagonist of Wnt/β-catenin signaling^15^, is expressed specifically in EKs, a Wnt/Dkk4 negative feedback loop has been proposed^26^. However, no morphological changes have been identified in the teeth of *Dkk4*-deficient mice^26,27^, leaving the role of *Dkk4* in EK patterning unclear.

Similar to *Dkk4*, *Notum* is a direct target of Wnt/β-catenin signaling and plays a crucial role as a Wnt inhibitor in a negative feedback loop, which is essential for *Drosophila* wing development and *Xenopus* brain development^1,2,28^. The role of Notum lies in inhibiting Wnt family members exclusively, and Notum stands out as the solitary secreted Wnt inhibitor found across the animal kingdom^1^. In the present study, we found *Notum* expression in primary and secondary EKs and the outer layer of dental papilla. *Notum-*deficient mice exhibited a notable size increase in secondary EKs in both maxillary and mandibular M1. The absence of *Notum* resulted in a significant elevation in the expression levels of Wnt pathway target genes, such as *Fgf20, Dkk4,* and *Fgf4,* in secondary EKs^26,29,30^. Intriguingly, these three EK marker genes showed a significant increase, while there was no notable change in the expression level of Wnt target genes in the mesenchyme. These findings collectively suggest that an activation in Wnt/β-catenin signaling, resulting from the suppression of inhibitors within the Wnt/Notum negative feedback loop, contributes to the enlargement of secondary EKs. The size of primary EK determines the number and size of cusps, as seen in previous studies on *Eda*-null mice or *c*^*IκBαΔN*^ mice, where a reduction in primary EK size resulted in decreased cusp number and size^14,23^. However, the impact of secondary EK size on the cusp and tooth dimensions remains unexplored. In this study, we found that the enlargement of secondary EKs led to broader cusp tips without altering the dimension of the cusp base in all molars in *Notum*-deficient mice. A potential mechanism through which the size of the secondary EK affects cusp tip dimensions involves Fgf signaling, known for its role in the remolding of cellular geometry. In gerbil molars, a linear expression pattern of *Fgf4* results in the long, flat lophs instead of cusps. Modifying Fgf signaling in gerbil tooth germs changes epithelial shape and epithelial invagination and transitions lophs to cusps^31^. In line with a previously identified regulatory module comprising Fgf4, Fgf20, RhoA, Rac1, and Cdh1, *Notum*-deficient mice displayed increased expression levels of *Fgf4* and *Fgf20.* However, the levels of *Rac1*, *Cdh1*, and *RhoA* did not change significantly, probably because these genes are expressed not only in EK but also in many other cells. These findings collectively suggest that Notum influences cusp tip dimensions by regulating the size of the secondary EK. Further investigations utilizing conditional *Notum*-deficient mice are imperative to ascertain whether the regulation of secondary EK size can be attributed to the epithelial Notum, mesenchymal Notum, or both. Previous studies have mainly explored the impact of mutations in *Shh*, *Sostdc1*, *Spry1*, *Spry2*, *Spry4*, *Rsk2*, *Eda*, *Edar*, and *Fgf3* on cusp number or tooth number in mice and humans^18,23,24,32–34^. Molars with changes in cusp arrangement without alterations in cusp number have been overlooked. In the present study, the maxillary M1 of *Notum*-deficient mice exhibited alterations in cusp arrangement but no discernible change in the overall cusp number. Displacement of many cusps in maxillary M1 of *Notum*-deficient mice had a direct impact on the crown outline. 

Consequently, *Notum*-deficient mice displayed a reduction of concavity in mesiolingual, mesiobuccal, and distolingual crown outlines in maxillary M1. The pattern of the crown outline exhibited a strong and direct relationship with the cusp pattern in maxillary M1. In mandibular M1 of *Notum*-deficient mice, though significant changes were observed in crown outline, a close relationship between the cusp pattern and the crown outline pattern was not identified since mandibular M1 of *Notum*-deficient M1 showed distinct scattering compared to wild-type M1 in PC analysis of cusp pattern.

Many literatures suggest a close interrelation between root and crown morphogenesis in mammalian teeth, given that root development consistently follows crown development^35–37^. Anomalies in crown morphology often coincide with irregularities in root morphology. For instance, human patients with *CACNA1S* or *WNT10A* mutations and *Wnt10a*-deficient mice show round-shaped molar crowns with root fusion and root taurodontism^38–41^. The cusp pattern and configuration of the crown outline are identified as pivotal factors in the shaping of cervical tongues, which subsequently determines the root pattern^42^. Consistent with findings from previous studies, we found that the cervical tongue was significantly shorter in areas where the concavity was reduced in the crown outline in maxillary M1 of *Notum*-deficient mice. Fusion between the short cervical tongues was delayed or failed, resulting in partial or complete root fusion. Furthermore, in our linear regression model, we once again confirmed a robust and direct relationship between the crown outline pattern and root pattern in both maxillary and mandibular M1.

**Figure 5:**
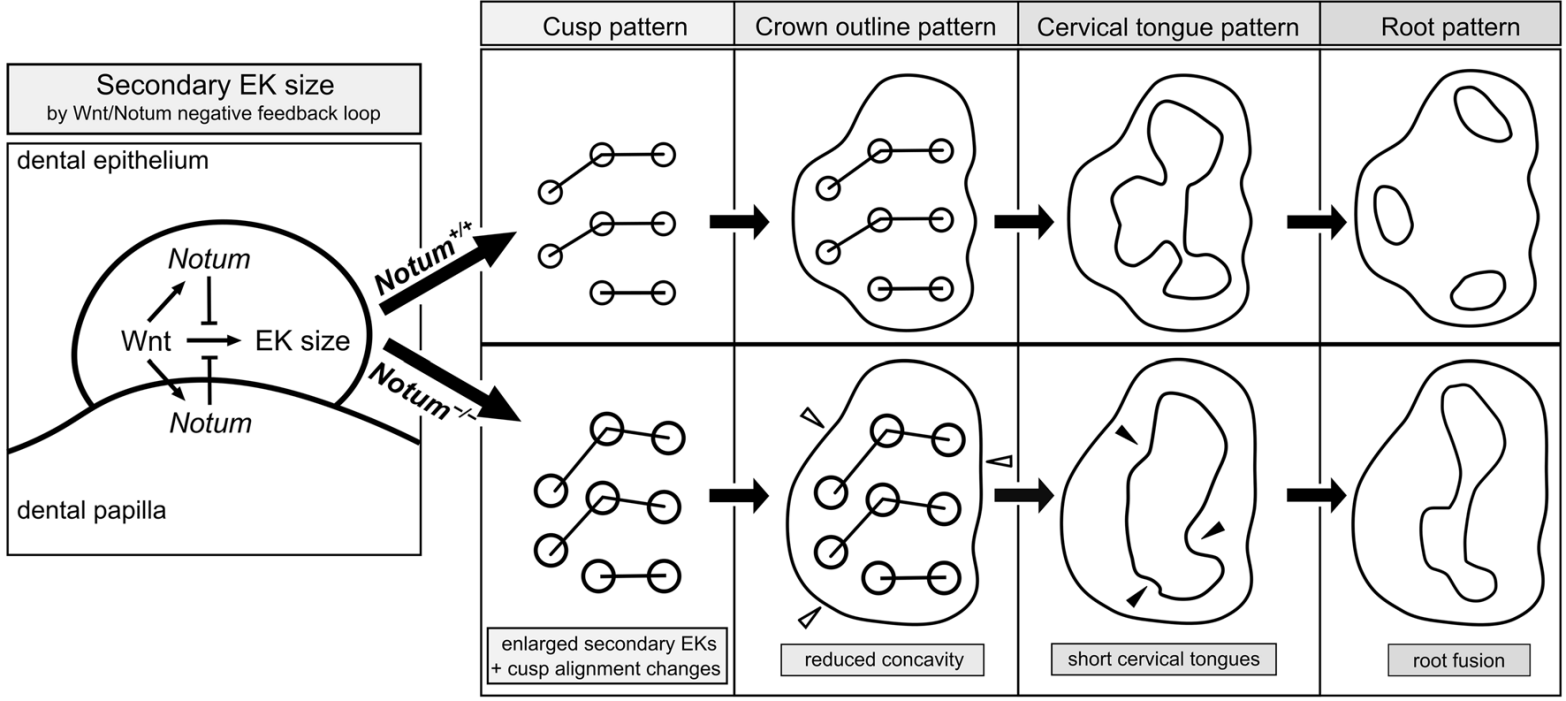
Schematic diagram illustrating *Notum* activity in the secondary enamel knot and its role in the crown and root patterning. *Notum* in secondary enamel knot (EK) and dental papilla regulates the size of secondary EK by inhibiting Wnt signaling. Loss of *Notum* leads to enlarged secondary EKs, resulting in broader cusp tips. Cusp displacement alters crown outline patterns, causing shorter cervical tongues. Subsequently, incomplete fusion between cervical tongues occurs, resulting in the incomplete separation of molar roots in *Notum*-deficient mice.

In summary, *Notum* expression was found in cells of primary EK, secondary EKs, and dental papilla during tooth development. The absence of *Notum* led to an enlargement of secondary EKs, contributing to broader cusp tips, suggesting that the secondary EK size is regulated through the Wnt/Notum negative feedback loop. The displacement of cusps in *Notum*-deficient mice resulted in changes in crown outline patterns, causing shorter cervical tongues. This, in turn, resulted in incomplete fusion between cervical tongues, causing an incomplete separation of molar roots in *Notum*-deficient mice (Fig. 5). These findings underscore the pivotal role of *Notum* in shaping both crown and root patterns by modulating the secondary EK size in the Wnt/Notum negative feedback loop.

### Supplementary Information

Supplementary Information.

## Data Availability

The publicly available single-cell RNA sequencing datasets analyzed in this study can be found at the National Center for Biotechnology Information (NCBI) Gene Expression Omnibus (GEO) under accession numbers GSE189381 and GSE162413. The raw and processed bulk RNA-Seq data associated with this study have been deposited in the NCBI GEO repository under the accession number GSE255946.
