# Supplementary material for: Notum regulates the cusp and root patterns in mouse molar
Source: Sci Rep. 2024 Jun 13;14:13633. doi: 10.1038/s41598-024-64340-w (PMC11176191; doi:10.1038/s41598-024-64340-w)
Supplement: Supplementary file 1 — Supplementary Information. [file 41598_2024_64340_MOESM1_ESM.pdf]

Notum Regulates the Cusp and Root Patterns in Mouse Molar

Dinuka Adasooriya[1], Ju-Kyung Jeong[2], Minjae Kyeong[1], Shiqi Kan[1], Jiwoo Kim[1], Eui-Sic Cho[2†], and Sung-Won Cho[1†]

[1]*Division of Anatomy and Developmental Biology, Department of Oral Biology, BK21 FOUR Project, Yonsei University College of Dentistry, Seoul, Korea*

[2]*Cluster for Craniofacial Development and Regeneration Research, Institute of Oral Biosciences, Jeonbuk National University School of Dentistry, Jeonju, Korea*

Supplementary Information

Including

Supplementary Figures

Supplementary Tables

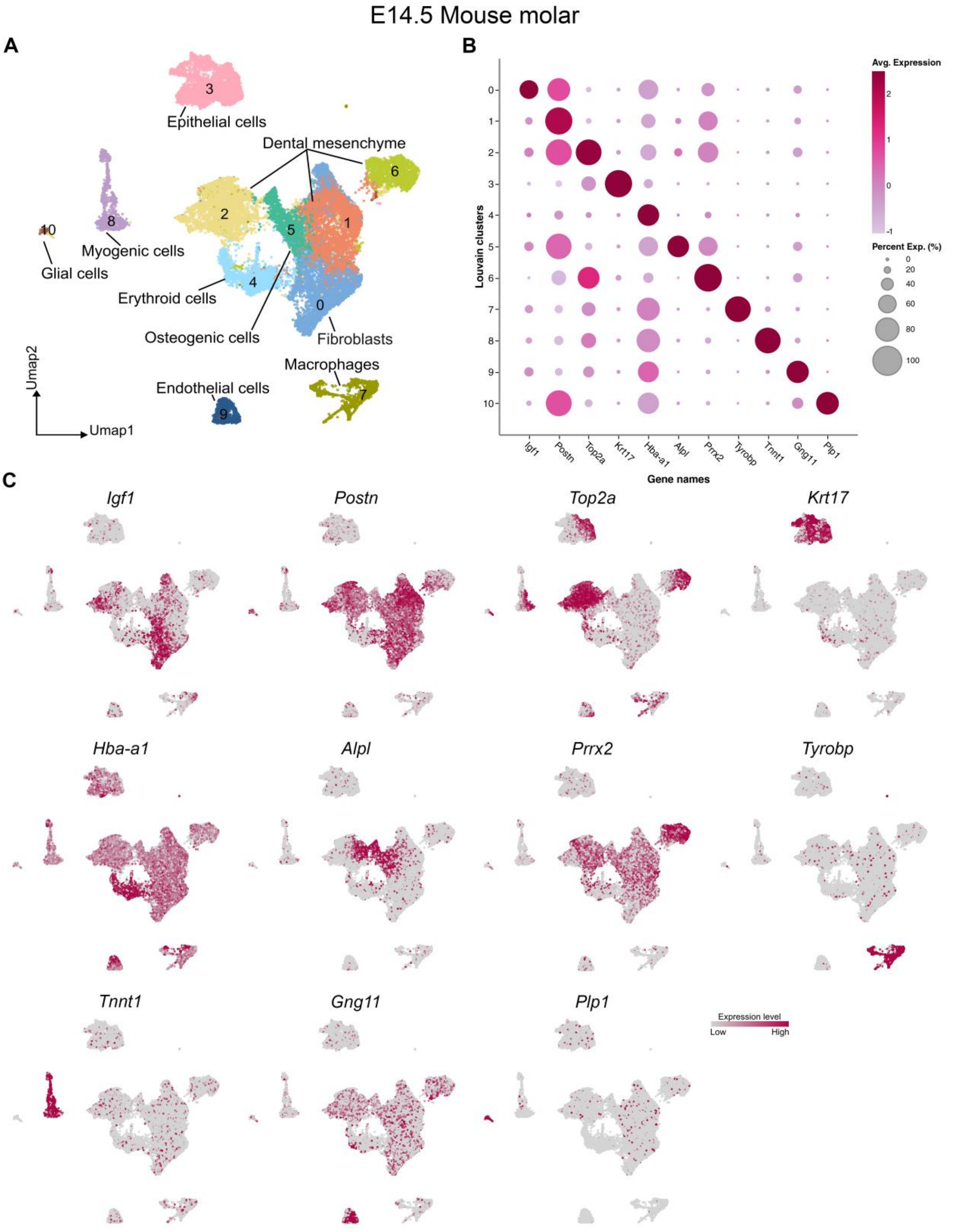

Supplementary Figure 1. Cell populations in developing mouse molars at E14.5. **(A)** An annotated UMAP plot with cell types in the mouse molar tooth germs at E14.5. **(B)** Dot plot demonstrating the scaled expression of selected signature genes in the cell populations shown in A. **(C)** UMAP feature plot of marker genes shown in dot plot B.

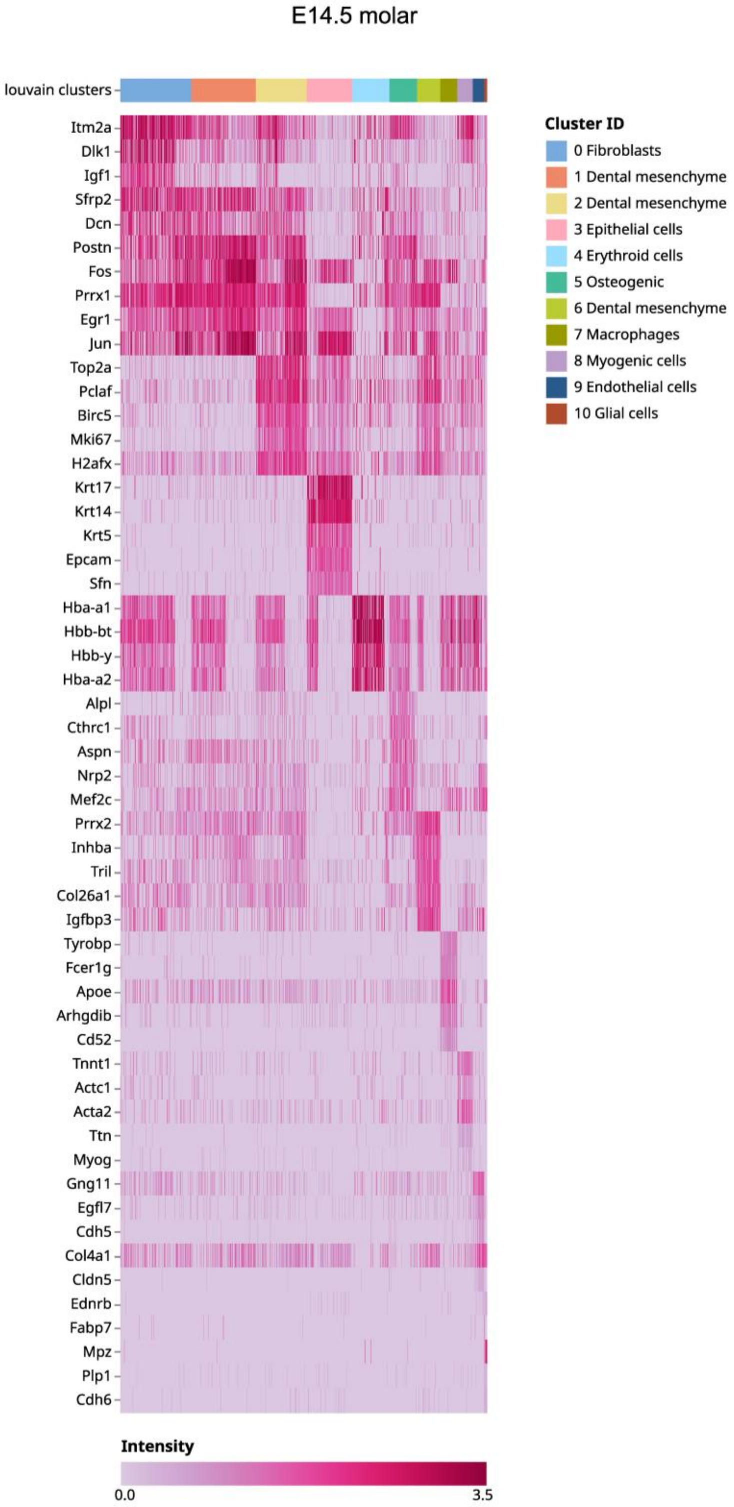

Supplementary Figure 2. Differential expression of genes between cell clusters in developing mouse molars at E14.5. Heatmap of top 5 differentially expressed genes between the Louvain clusters in the mouse molar tooth germs at E14.5.

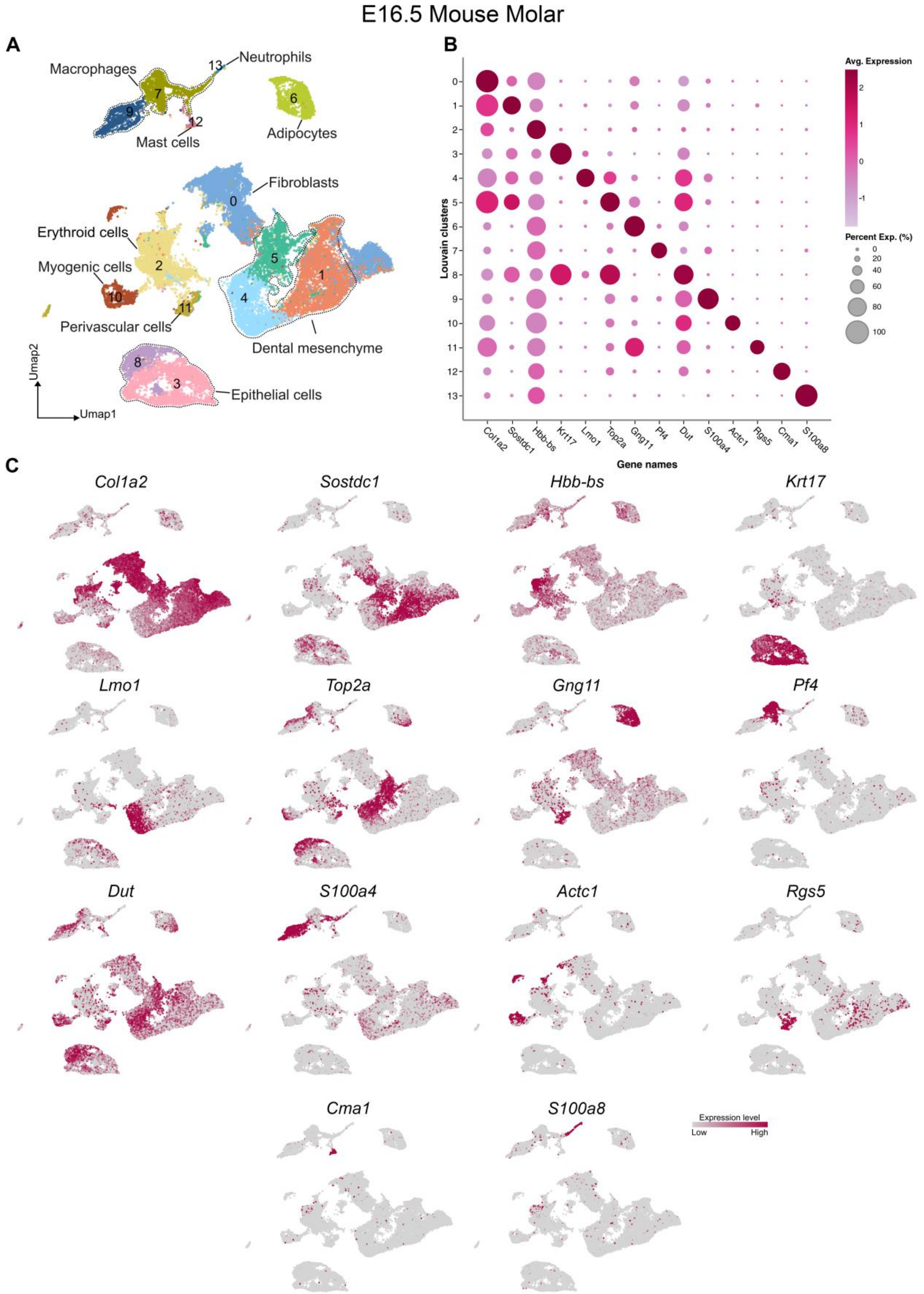

Supplementary Figure 3. Cell populations in developing mouse molars at E16.5. **(A)** An annotated UMAP plot with cell types in the mouse molar tooth germs with at E16.5. **(B)** Dot plot demonstrating the scaled expression of selected signature genes in the cell populations shown in A. **(C)** UMAP feature plot of marker genes shown in dot plot B.

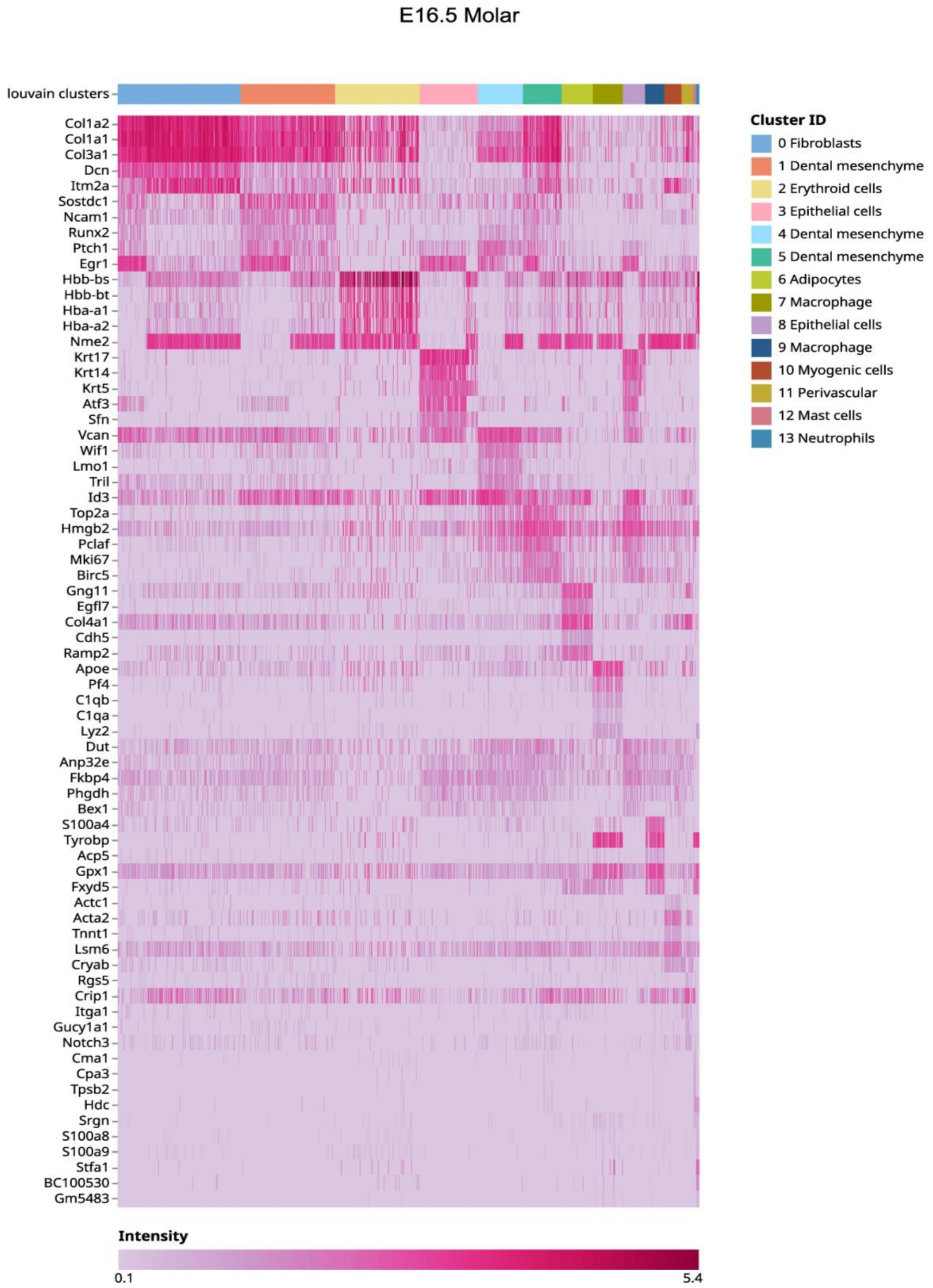

Supplementary Figure 4. Differential expression of genes between cell clusters in developing mouse molars at E16.5. Heatmap of top 5 differentially expressed genes between the Louvain clusters in the mouse molar tooth germs at E16.5

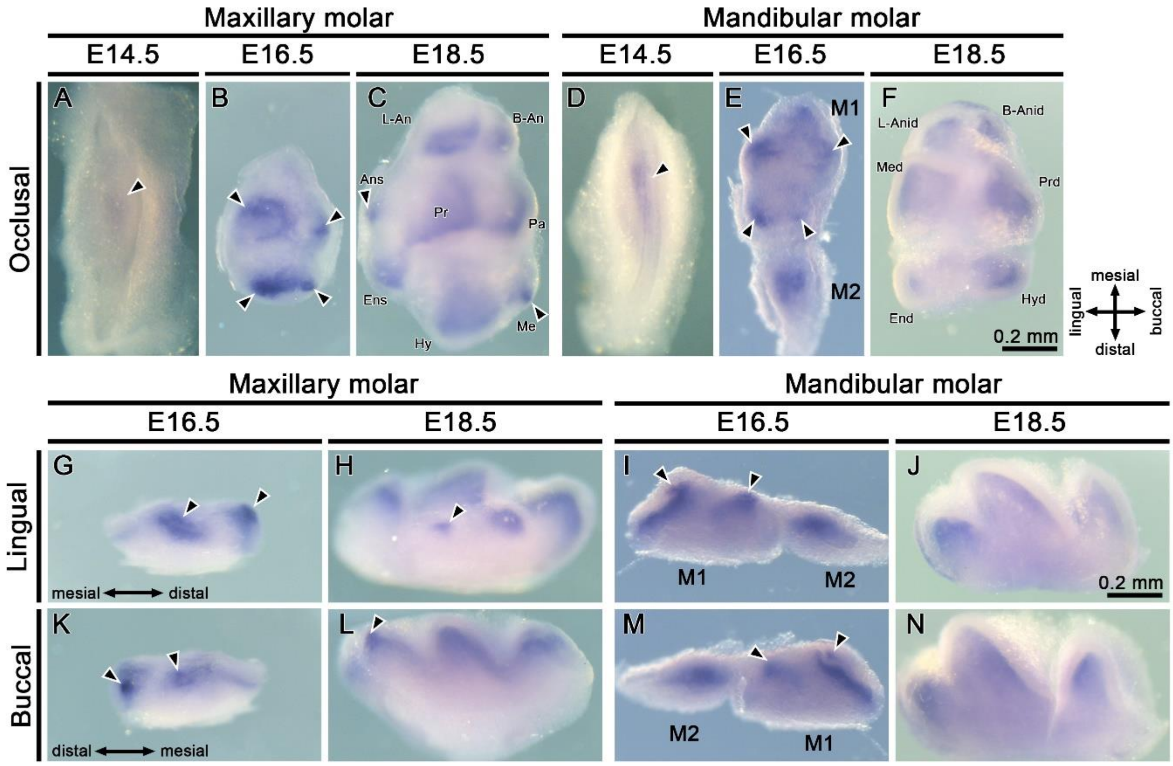

Supplementary Figure 5. *Notum* expression in developing molars in the maxilla and mandible. **(A-F)** *Notum* expression (arrowheads) in molars from an occlusal view. *Notum* is initially expressed in the center of the first molar (M1) at E14.5 and subsequently in the cusps at E16.5 and E18.5. **(G-N)** *Notum* expression in molars from both the lingual and buccal views. At E16.5, the epithelial *Notum* expression (arrowheads) is observed in the majority of M1 cusps. However, at E18.5, this epithelial *Notum* expression is limited to a few cusps that formed relatively late. Mesenchymal *Notum* expression is evident in the outer layer of the dental papilla at both E16.5 and E18.5.

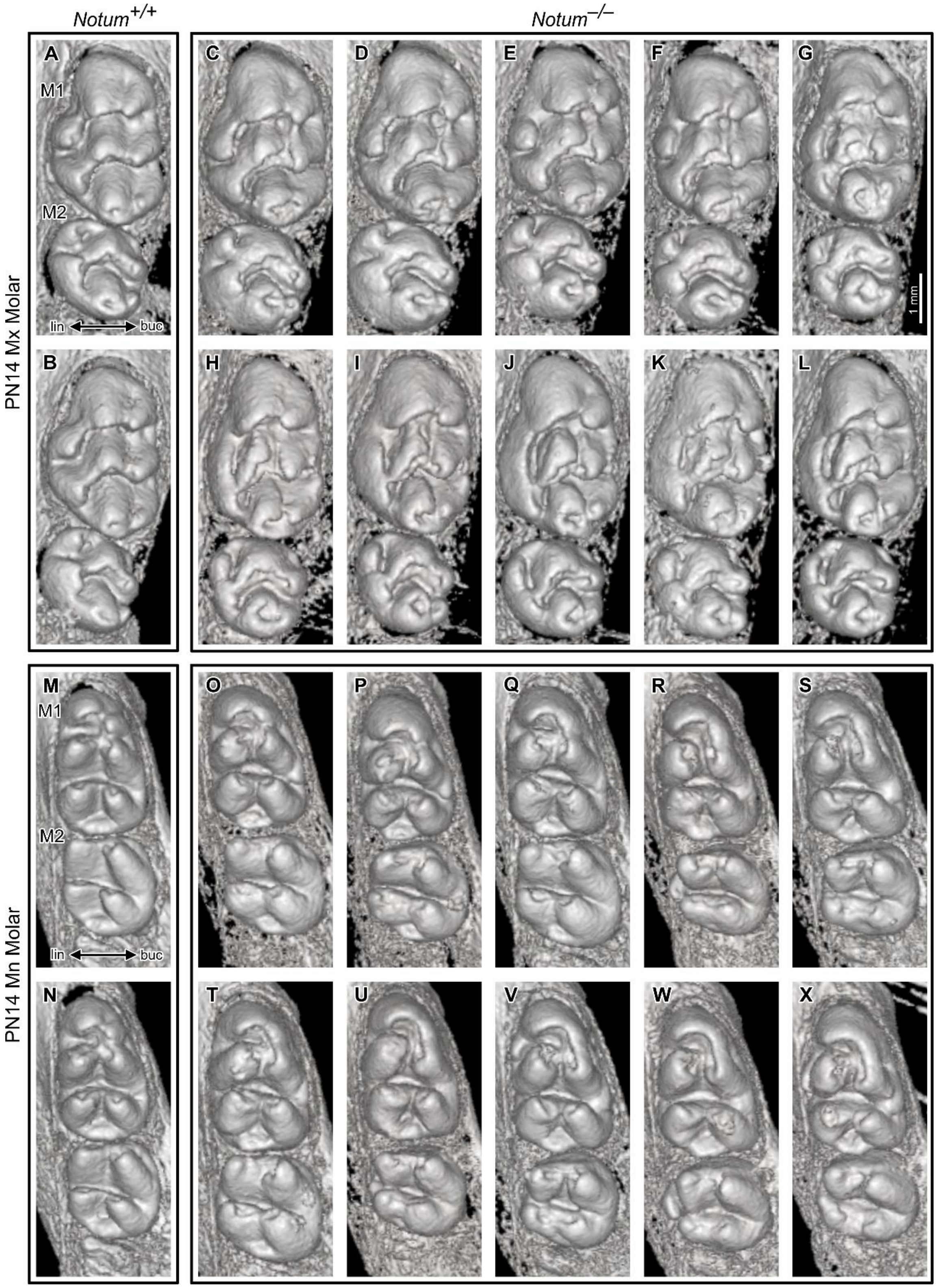

Supplementary Figure 6. Morphological variations in crown of maxillary and mandibular molars of *Notum*$^{-/-}$ mice at PN 14. **(A−B)** Occlusal view of maxillary molars in *Notum*$^{+/+}$ mice. **(C−L)** Occlusal view of maxillary molars in *Notum*$^{-/-}$ mice. *Notum*$^{-/-}$ M1 molars show broader cusp tips compared to the *Notum*$^{+/+}$ mice. **(M−N)** Occlusal view of mandibular molars in *Notum*$^{+/+}$ mice. **(O−X)** Occlusal view of maxillary molars in *Notum*$^{-/-}$ mice. *Notum*$^{-/-}$ mice showed the fusion between anterostyle and enterostyle in maxillary M1s (H−L) and the fusion between lingual anteroconid, buccal anteroconid, and protoconid in most of mandibular M1s (R−X).

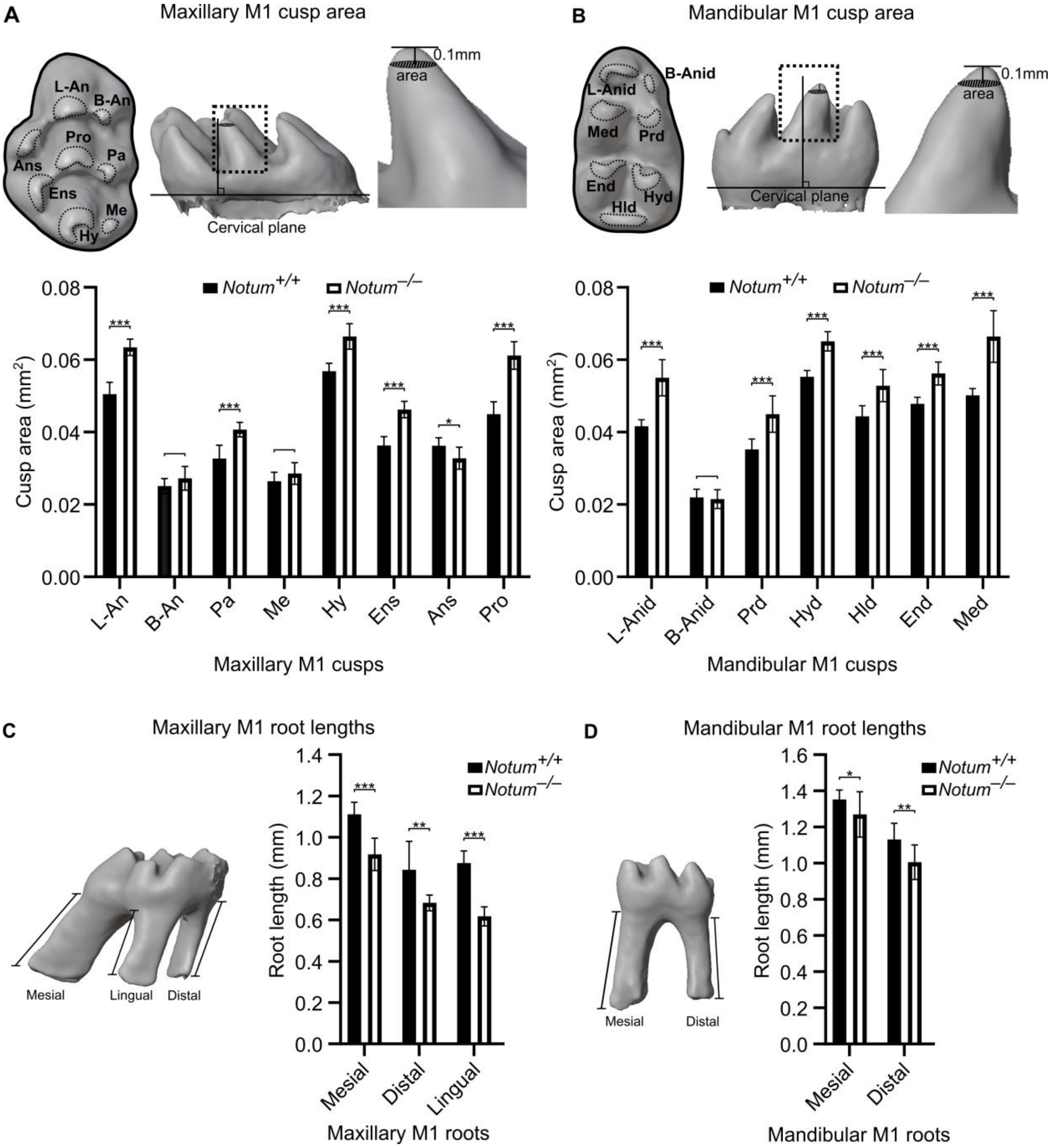

Supplementary Figure 7. Changes in cusp tip area and root length in *Notum*$^{-/-}$ M1 molars. **(A−B)** *Notum*$^{-/-}$ mice show a significant increase in cusp tip area in the majority of the maxillary except the buccal anterocone, and metacone and in the mandibular cusps except the buccal anteroconid (n = 10 for each group). **(C−D)** *Notum*$^{-/-}$ mice show significantly shorter roots in maxillary and mandibular M1 compared to the *Notum*$^{-/-}$ mice (n = 10 for each group). L-An: lingual anterocone, B-An: buccal anterocone, Pa: paracone, Me: metacone, Hy: hypocone, Ens: enterostyle, Ans: anterostyle, Pr: protocone, L-Anid: lingual anteroconid, B-Anid: buccal anteroconid, Prd: protoconid, Hyd: hypoconid, Hld: hypoconulid. End: entoconid, Med: metaconid. Mann Whitney U test, *P < 0.05, **P < 0.01 and ***P < 0.001.

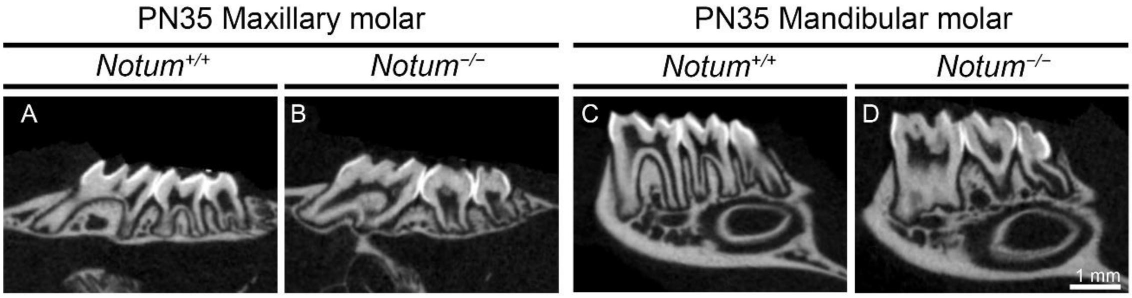

Supplementary Figure 8. Maxillary and mandibular molars appearance in micro-CT sections. **(A-D)** The phenotypes in molars of PN35 *Notum*$^{+/+}$ and *Notum*$^{-/-}$ mice. The sagittal sections show that the maxillary and the mandibular M1 and M2 roots are fused together in *Notum*$^{-/-}$ compared to the *Notum*$^{+/+}$ mice. The enamel shows a bright white color with similar intensity and thickness in *Notum*$^{+/+}$ and *Notum*$^{-/-}$ molars. *Notum*$^{-/-}$ molars show severe attrition compared to the *Notum*$^{+/+}$ molars. As a result, the enamel is absent on the occlusal surface.

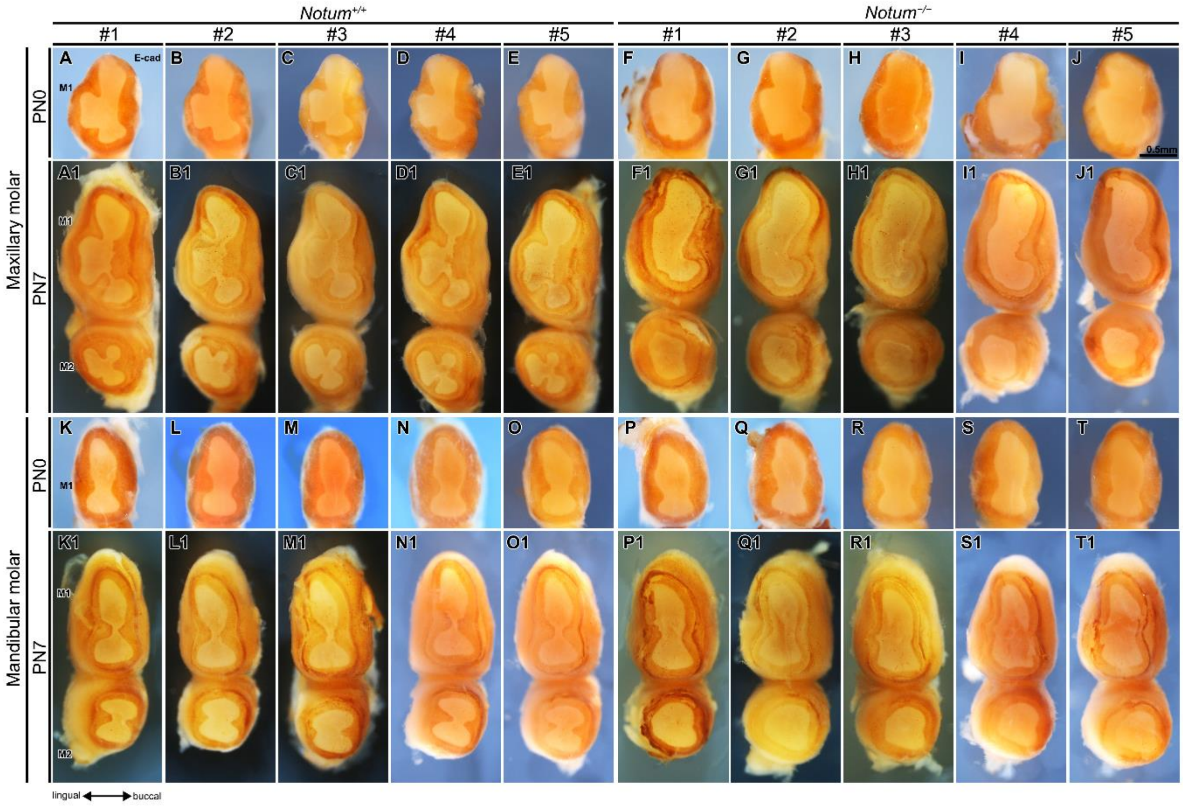

Supplementary Figure 9. Altered cervical tongue morphology of *Notum*$^{-/-}$ molars**. (A-T1)** The localization of the E-cadherin in the apical view of maxillary and mandibular M1 at PN0 and PN7 (n = 5 per group). *Notum*$^{-/-}$ M1s display shorter cervical tongues and wider gaps between cervical tongues compared to *Notum*$^{+/+}$ M1.

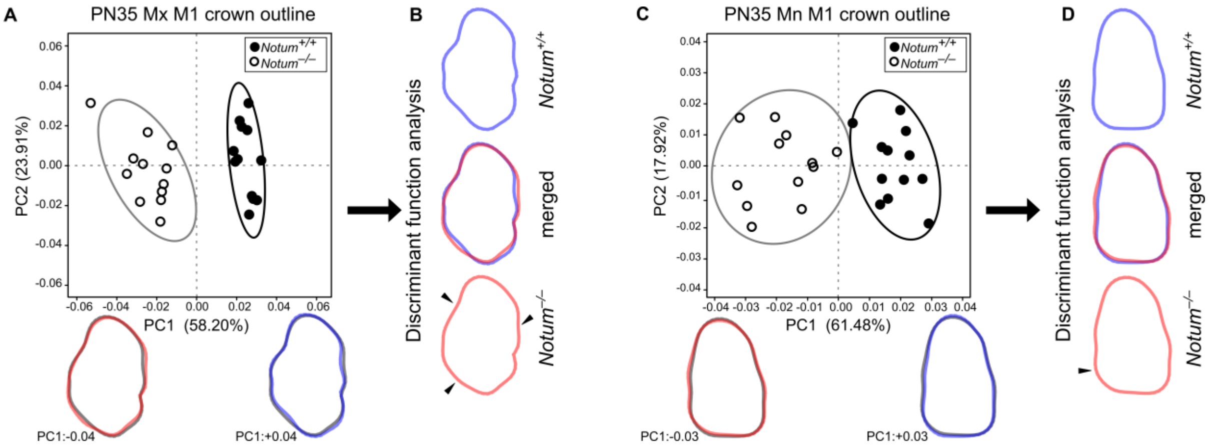

Supplementary Figure 10. Geometric morphometric changes in cusp pattern and crown outline in *Notum*$^{-/-}$ mice at PN35. **(A–D)** PCA of maxillary and mandibular crown outline at PN 35 (n = 12 for each group). *Notum*$^{-/-}$ M1s are clustered separately from the *Notum*$^{+/+}$ M1 on the PC1 axis. Cross-validated DF analysis correctly classifies *Notum*$^{-/-}$ M1 into *Notum*$^{-/-}$ M1 group and *Notum*$^{+/+}$ M1 as *Notum*$^{+/+}$ M1 group with 100% predictive accuracy in the maxilla. In mandible. *Notum*$^{-/-}$ M1s are correctly classified into *Notum*$^{-/-}$ M1 group with 92% predictive accuracy and *Notum*$^{+/+}$ M1 into *Notum*$^{+/+}$ M1 group with 100% predictive accuracy. Morphometric changes in crown outline at PN35 mirror those at PN14 (arrowheads in B, D).

Supplementary Table 1. Top 50 upregulated genes in molars of *Notum*$^{-/-}$ mice compared to *Notum*$^{+/+}$ mice at E14.5 (fold change > 2, FPKM > 0.3)

Gene id	Gene name	Gene description	P value	Fold change
ENSMUSG00000044041	*Krt13*	keratin 13	1.6731E-196	8.472408724
ENSMUSG00000033825	*Tpsb2*	tryptase beta 2	8.54034E-08	6.862759807
ENSMUSG00000050578	*Mmp13*	matrix metallopeptidase 13	2.75126E-16	6.530235624
ENSMUSG00000059668	*Krt4*	keratin 4	3.30006E-71	5.759029893
ENSMUSG00000062694	*Cav3*	caveolin 3	1.73517E-10	4.868339895
ENSMUSG00000034634	*Ly6d*	lymphocyte antigen 6 complex, locus D	2.46197E-12	4.561926433
ENSMUSG00000052974	*Cyp2f2*	cytochrome P450, family 2, subfamily f, polypeptide 2	1.12626E-10	4.296644118
ENSMUSG00000078787	*Cyp2t4*	cytochrome P450, family 2, subfamily t, polypeptide 4	4.24759E-08	4.167150112
ENSMUSG00000022225	*Cma1*	chymase 1, mast cell	1.31986E-13	3.810918215
ENSMUSG00000030399	*Ckm*	creatine kinase, muscle	8.02863E-13	3.809788332
ENSMUSG00000050359	*Sprr1a*	small proline-rich protein 1A	0.013552525	3.682952995
ENSMUSG00000032484	*Ngp*	neutrophilic granule protein	0.000257532	3.634824115
ENSMUSG00000022902	*Stfa2*	stefin A2	0.028697929	3.591219906
ENSMUSG00000016327	*Atp1b4*	ATPase, (Na+)/K+ transporting, beta 4 polypeptide	2.3677E-14	3.553799041
ENSMUSG00000041476	*Smpx*	small muscle protein, X-linked	6.60277E-06	3.544493618
ENSMUSG00000061723	*Tnnt3*	troponin T3, skeletal, fast	1.28332E-18	3.533313363
ENSMUSG00000038086	*Hspb2*	heat shock protein 2	0.003232214	3.406613524
ENSMUSG00000020836	*Coro6*	coronin 6	1.63957E-05	3.288319163
ENSMUSG00000020067	*Mypn*	myopalladin	8.28225E-09	3.092999296

ENSMUSG00000027360	*Hdc*	histidine decarboxylase	3.7147E-08	3.068094229
ENSMUSG00000021798	*Ldb3*	LIM domain binding 3	8.02393E-23	2.983968723
ENSMUSG00000079588	*Tmem182*	transmembrane protein 182	0.000210177	2.900610777
ENSMUSG00000040666	*Sh3bgr*	SH3-binding domain glutamic acid-rich protein	0.002015265	2.897293432
ENSMUSG00000030785	*Cox6a2*	cytochrome c oxidase subunit 6A2	0.000210538	2.859188116
ENSMUSG00000044086	*Lmod3*	leiomodin 3 (fetal)	7.38224E-07	2.854652347
ENSMUSG00000056071	*S100a9*	S100 calcium binding protein A9 (calgranulin B)	3.0686E-05	2.822510872
ENSMUSG00000033044	*Dhrs7c*	dehydrogenase/reductase (SDR family) member 7C	0.010093723	2.798812036
ENSMUSG00000078815	*Cacng6*	calcium channel, voltage-dependent, gamma subunit 6	0.00147539	2.788687603
ENSMUSG00000063130	*Calml3*	calmodulin-like 3	1.48179E-05	2.7212056
ENSMUSG00000079679	*Vwde*	von Willebrand factor D and EGF domains	7.84061E-08	2.709865461
ENSMUSG00000027861	*Casq2*	calsequestrin 2	1.26144E-18	2.683233703
ENSMUSG00000031972	*Acta1*	actin, alpha 1, skeletal muscle	2.23828E-61	2.674285926
ENSMUSG00000049641	*Vgll2*	vestigial like family member 2	8.58412E-09	2.666943116
ENSMUSG00000020722	*Cacng1*	calcium channel, voltage-dependent, gamma subunit 1	0.00394124	2.640780085
ENSMUSG00000031097	*Tnni2*	troponin I, skeletal, fast 2	4.98489E-07	2.634856831
ENSMUSG00000020676	*Ccl11*	chemokine (C-C motif) ligand 11	0.040551527	2.633279088
ENSMUSG00000026407	*Cacna1s*	calcium channel, voltage-dependent, L type, alpha 1S subunit	9.80383E-08	2.596296865
ENSMUSG00000024471	*Myot*	myotilin	5.32683E-07	2.589819769
ENSMUSG00000020061	*Mybpc1*	myosin binding protein C, slow-type	3.56055E-25	2.588082671
ENSMUSG00000079428	*Tceal7*	transcription elongation factor A (SII)-like 7	2.10282E-11	2.581518632
ENSMUSG00000028116	*Myoz2*	myozenin 2	5.94744E-05	2.576291734
ENSMUSG00000047819	*Tigd4*	tigger transposable element derived 4	0.001540891	2.576152854
ENSMUSG00000060913	*Trim55*	tripartite motif-containing 55	2.29574E-09	2.569595539
ENSMUSG00000030672	*Mylpf*	myosin light chain, phosphorylatable, fast skeletal muscle	1.14324E-31	2.545394725
ENSMUSG00000026253	*Chrng*	cholinergic receptor, nicotinic, gamma polypeptide	2.66428E-10	2.519904464
ENSMUSG00000055775	*Myh8*	myosin, heavy polypeptide 8, skeletal muscle, perinatal	3.71311E-27	2.509829235
ENSMUSG00000001508	*Sgca*	sarcoglycan, alpha (dystrophin-associated glycoprotein)	0.000633746	2.495104807
ENSMUSG00000026414	*Tnnt2*	troponin T2, cardiac	1.58098E-18	2.494283998
ENSMUSG00000022215	*Fitm1*	fat storage-inducing transmembrane protein 1	0.009040807	2.490344662
ENSMUSG00000001865	*Cpa3*	carboxypeptidase A3, mast cell	2.08137E-10	2.418955725

Supplementary Table 2. Downregulated genes in molars of *Notum*$^{-/-}$ mice compared to *Notum*$^{+/+}$ mice at E14.5 (fold change > 2, FPKM > 0.3)

Gene id	Gene name	Gene description	P value	Fold change
ENSMUSG00000004366	*Sst*	somatostatin	0.005212	-6.50524747
ENSMUSG00000079466	*Prdm12*	PR domain containing 12	0.00707	-5.96851805
ENSMUSG00000014603	*Alx3*	aristaless-like homeobox 3	3.13E-07	-5.72628632
ENSMUSG00000038193	*Hand2*	heart and neural crest derivatives expressed 2	3.63E-14	-5.21463018
ENSMUSG00000032357	*Tinag*	tubulointerstitial nephritis antigen	0.021886	-4.95707814
ENSMUSG00000001494	*Sost*	sclerostin	0.002211	-4.86456741
ENSMUSG00000036602	*Alx1*	ALX homeobox 1	3.56E-21	-4.72369957
ENSMUSG00000058626	*Capn11*	calpain 11	2.08E-07	-4.5135441
ENSMUSG00000022212	*Cpne6*	copine VI	0.003385	-4.23600858
ENSMUSG00000004872	*Pax3*	paired box 3	1.96E-07	-3.69784715
ENSMUSG00000036437	*Npy1r*	neuropeptide Y receptor Y1	0.004502	-2.75407373
ENSMUSG00000053024	*Cntn2*	contactin 2	4.44E-06	-2.58152529
ENSMUSG00000032318	*Isl2*	insulin related protein 2 (islet 2)	0.027131	-2.41925863
ENSMUSG00000042988	*Notum*	notum palmitoleoyl-protein carboxylesterase	0.000756	-2.21190188
ENSMUSG00000020018	*Snrpf*	small nuclear ribonucleoprotein polypeptide F	5.01E-06	-2.14008280
ENSMUSG00000027071	*P2rx3*	purinergic receptor P2X, ligand-gated ion channel, 3	0.034761	-2.06319196
ENSMUSG00000028072	*Ntrk1*	neurotrophic tyrosine kinase, receptor, type 1	0.001093	-2.03138351

Supplementary Table 3. Upregulated genes in molars of *Notum*$^{-/-}$ mice compared to *Notum*$^{+/+}$ mice at E16.5 (fold change > 2, FPKM > 0.3)

Gene id	Gene name	Gene description	P value	Fold change
ENSMUSG00000019932	*Kera*	keratocan	7.13E-48	5.714947
ENSMUSG00000031603	*Fgf20*	fibroblast growth factor 20	2.07E-06	4.373358
ENSMUSG00000064360	*mt-Nd3*	mitochondrially encoded NADH dehydrogenase 3	2.84E-13	3.917385
ENSMUSG00000021622	*Ckmt2*	creatine kinase, mitochondrial 2	1.21E-08	3.555372
ENSMUSG00000068122	*Agtr2*	angiotensin II receptor, type 2	1.81E-100	3.052717
ENSMUSG00000064358	*mt-Co3*	mitochondrially encoded cytochrome c oxidase III	4.98E-07	3.024279
ENSMUSG00000059108	*Ifitm6*	interferon induced transmembrane protein 6	0.033944	2.974692
ENSMUSG00000063245	*Zfp993*	zinc finger protein 993	0.000471	2.958191
ENSMUSG00000042254	*Cilp*	cartilage intermediate layer protein, nucleotide pyrophosphohydrolase	1.94E-11	2.925638
ENSMUSG00000001865	*Cpa3*	carboxypeptidase A3, mast cell	3.26E-16	2.608288
ENSMUSG00000056643	*Chst13*	carbohydrate (chondroitin 4) sulfotransferase 13	0.031958	2.538128
ENSMUSG00000022225	*Cma1*	chymase 1, mast cell	9.36E-19	2.460558
ENSMUSG00000053093	*Myh7*	myosin, heavy polypeptide 7, cardiac muscle, beta	1.11E-18	2.433521

ENSMUSG00000010797	*Wnt2*	wingless-type MMTV integration site family, member 2	0.010194	2.381359
ENSMUSG00000025044	*Msr1*	macrophage scavenger receptor 1	8.28E-05	2.339884
ENSMUSG00000031535	*Dkk4*	dickkopf WNT signaling pathway inhibitor 4	3.33E-05	2.332174
ENSMUSG00000032419	*Tbx18*	T-box18	8.37E-07	2.278756
ENSMUSG00000070385	*Ampd1*	adenosine monophosphate deaminase 1	2.61E-07	2.256862
ENSMUSG00000092035	*Peg10*	paternally expressed 10	4.59E-71	2.245729
ENSMUSG00000035258	*Abi3bp*	ABI gene family, member 3 (NESH) binding protein	4.70E-42	2.243991
ENSMUSG00000064354	*mt-Co2*	mitochondrially encoded cytochrome c oxidase II	6.84E-08	2.241564
ENSMUSG00000052276	*Ostn*	osteocrin	0.002977	2.214417
ENSMUSG00000054293	*P2ry10b*	purinergic receptor P2Y, G-protein coupled 10B	4.24E-05	2.208457
ENSMUSG00000049115	*Agtr1a*	angiotensin II receptor, type 1a	6.41E-05	2.202085
ENSMUSG00000052854	*Nrk*	Nik related kinase	2.57E-57	2.194555
ENSMUSG00000024529	*Lox*	lysyl oxidase	2.57E-93	2.182619
ENSMUSG00000005220	*Corin*	corin	4.78E-08	2.161709
ENSMUSG00000019787	*Trdn*	triadin	7.28E-05	2.128557
ENSMUSG00000020805	*Slc13a5*	solute carrier family 13 (sodium-dependent citrate transporter), member 5	6.74E-10	2.121354
ENSMUSG00000022595	*Lypd2*	Ly6/Plaur domain containing 2	0.035254	2.112494
ENSMUSG00000050931	*Sgms2*	sphingomyelin synthase 2	3.55E-32	2.087137
ENSMUSG00000027559	*Car3*	carbonic anhydrase 3	2.71E-16	2.075139
ENSMUSG00000021390	*Ogn*	osteoglycin	1.23E-70	2.070199
ENSMUSG00000019817	*Plagl1*	pleiomorphic adenoma gene-like 1	1.63E-109	2.065845
ENSMUSG00000074934	*Grem1*	gremlin 1, DAN family BMP antagonist	0.000162	2.060451
ENSMUSG00000036144	*Meox2*	mesenchyme homeobox 2	3.71E-09	2.055837
ENSMUSG00000051747	*Ttn*	titin	4.81E-67	2.052107
ENSMUSG00000042607	*Asb4*	ankyrin repeat and SOCS box-containing 4	2.53E-10	2.04622
ENSMUSG00000042045	*Sln*	sarcolipin	2.83E-12	2.038246
ENSMUSG00000045613	*Chrm2*	cholinergic receptor, muscarinic 2, cardiac	5.98E-06	2.031555
ENSMUSG00000022342	*Kcnv1*	potassium channel, subfamily V, member 1	2.09E-05	2.027014
ENSMUSG00000036855	*Gjd4*	gap junction protein, delta 4	0.001773	2.024189
ENSMUSG00000020077	*Srgn*	serglycin	0.000572	2.022666
ENSMUSG00000026586	*Prrx1*	paired related homeobox 1	1.30E-89	2.022269
ENSMUSG00000021388	*Aspn*	asporin	1.55E-72	2.016374

Supplementary Table 4. Top 50 downregulated genes in molars of *Notum*$^{-/-}$ mice compared to *Notum*$^{+/+}$ mice at E16.5 (fold change > 2, FPKM > 0.3)

Gene id	Gene name	Gene description	P value	Fold change
ENSMUSG00000079466	*Prdm12*	PR domain containing 12	5.07E-07	-236.839
ENSMUSG00000042448	*Hoxd1*	homeobox D1	2.05E-06	-192.528

ENSMUSG00000091519	*Skor2*	SKI family transcriptional corepressor 2	3.01E-06	-181.868
ENSMUSG00000029608	*Rph3a*	rabphilin 3A	2.80E-11	-145.677
ENSMUSG00000030500	*Slc17a6*	solute carrier family 17 (sodium-dependent inorganic phosphate cotransporter), member 6	3.44E-11	-143.896
ENSMUSG00000070552	*Mrgprx1*	MAS-related GPR, member X1	2.08E-05	-134.673
ENSMUSG00000005994	*Tyrp1*	tyrosinase-related protein 1	2.05E-06	-127.625
ENSMUSG00000024497	*Pou4f3*	POU domain, class 4, transcription factor 3]	4.55E-05	-118.419
ENSMUSG00000060257	*Scrt2*	scratch family zinc finger 2	5.19E-06	-109.476
ENSMUSG00000048385	*Scrt1*	scratch family zinc finger 1	8.39E-10	-105.039
ENSMUSG00000022212	*Cpne6*	copine VI	3.60E-20	-87.2067
ENSMUSG00000034533	*Scn10a*	sodium channel, voltage-gated, type X, alpha	4.98E-12	-78.5396
ENSMUSG00000033615	*Cplx1*	complexin 1	9.54E-35	-63.6583
ENSMUSG00000034683	*Ppp1r1c*	protein phosphatase 1, regulatory inhibitor subunit 1C	1.13E-10	-42.6793
ENSMUSG00000048483	*Zdhhc22*	zinc finger, DHHC-type containing 22	6.51E-06	-42.28
ENSMUSG00000020396	*Nefh*	neurofilament, heavy polypeptide	7.82E-59	-39.1087
ENSMUSG00000022054	*Nefm*	neurofilament, medium polypeptide	4.67E-54	-37.0878
ENSMUSG00000023064	*Sncg*	synuclein, gamma	8.34E-26	-26.0073
ENSMUSG00000020704	*Asic2*	acid-sensing (proton-gated) ion channel 2	4.44E-07	-20.0598
ENSMUSG00000027581	*Stmn3*	stathmin-like 3	1.56E-46	-19.9989
ENSMUSG00000046814	*Gchfr*	GTP cyclohydrolase I feedback regulator	0.001607	-19.0053
ENSMUSG00000016346	*Kcnq2*	potassium voltage-gated channel, subfamily Q, member 2	6.42E-24	-18.9848
ENSMUSG00000034891	*Sncb*	synuclein, beta	1.70E-05	-18.5521
ENSMUSG00000075402	*Krt76*	keratin 76	0.001879	-18.493
ENSMUSG00000030302	*Atp2b2*	ATPase, Ca++ transporting, plasma membrane 2	7.33E-21	-17.8797
ENSMUSG00000022055	*Nefl*	neurofilament, light polypeptide	6.64E-156	-17.6702
ENSMUSG00000031762	*Mt2*	metallothionein 2	0.00453	-16.9501
ENSMUSG00000021194	*Chga*	chromogranin A	1.77E-15	-16.2079
ENSMUSG00000027071	*P2rx3*	purinergic receptor P2X, ligand-gated ion channel, 3	5.20E-28	-15.4759
ENSMUSG00000048349	*Pou4f1*	POU domain, class 4, transcription factor 1	3.65E-13	-15.4624
ENSMUSG00000030854	*Ptpn5*	protein tyrosine phosphatase, non-receptor type 5	3.60E-08	-15.4442
ENSMUSG00000054325	*Lce3a*	late cornified envelope 3A	0.013181	-15.2098
ENSMUSG00000037843	*Vstm2l*	V-set and transmembrane domain containing 2-like	6.52E-15	-13.6106
ENSMUSG00000037428	*Vgf*	VGF nerve growth factor inducible	5.56E-10	-12.635
ENSMUSG00000031144	*Syp*	synaptophysin	1.21E-25	-12.6099
ENSMUSG00000070570	*Slc17a7*	solute carrier family 17 (sodium-dependent inorganic phosphate cotransporter),	3.48E-08	-11.8672

		member 7		
ENSMUSG00000040610	*Tlx3*	T cell leukemia, homeobox 3	0.001867	-11.6593
ENSMUSG00000040907	*Atp1a3*	ATPase, Na+/K+ transporting, alpha 3 polypeptide	2.62E-61	-11.5879
ENSMUSG00000018411	*Mapt*	microtubule-associated protein tau	1.78E-57	-11.1714
ENSMUSG00000023484	*Prph*	peripherin	9.82E-105	-11.0236
ENSMUSG00000039809	*Gabbr2*	gamma-aminobutyric acid (GABA) B receptor, 2	3.28E-17	-10.9133
ENSMUSG00000032318	*Isl2*	insulin related protein 2 (islet 2)	7.68E-09	-10.7512
ENSMUSG00000026347	*Tmem163*	transmembrane protein 163	1.37E-07	-10.6614
ENSMUSG00000025432	*Avil*	advillin	1.10E-36	-10.4753
ENSMUSG00000036437	*Npy1r*	neuropeptide Y receptor Y1	8.78E-07	-10.0518
ENSMUSG00000033061	*Resp18*	regulated endocrine-specific protein 18	0.000771	-9.09128
ENSMUSG00000038916	*Soga3*	SOGA family member 3	1.42E-08	-9.0648
ENSMUSG00000068885	*Lce3f*	late cornified envelope 3F	0.000214	-8.78343
ENSMUSG00000039278	*Pcsk1n*	proprotein convertase subtilisin/kexin type 1 inhibitor	2.95E-20	-8.70905
ENSMUSG00000056569	*Mpz*	myelin protein zero	3.67E-76	-8.50579
